# RUNX1 contributes to the mesenchymal subtype of glioblastoma in a TGFβ pathway-dependent manner

**DOI:** 10.1038/s41419-019-2108-x

**Published:** 2019-11-21

**Authors:** Kai Zhao, Xiaoteng Cui, Qixue Wang, Chuan Fang, Yanli Tan, Yunfei Wang, Kaikai Yi, Chao Yang, Hua You, Rui Shang, Jiachong Wang, Chunsheng Kang

**Affiliations:** 1 Lab of Neuro-oncology, Tianjin Neurological Institute, Key Laboratory of Post-Neuroinjury Neuro-repair and Regeneration in Central Nervous System, Department of Neurosurgery, Tianjin Medical University General Hospital, Tianjin, China; 2 Department of Neurosurgery, the Affiliated Hospital of Qingdao University, Qingdao, China; 3 Department of Neurosurgery, Affiliated hospital of Hebei University, Baoding, China; 4 Department of Pathology, Affiliated hospital of Hebei University, Baoding, China; 5 Department of Oncology, Affiliated Cancer Hospital & Institute of Guangzhou Medical University, Guangzhou, China; 6 Department of Radiation Oncology, Weihai Municipal Hospital, Weihai, China; 7 Department of Neurosurgery, Affiliated Haikou Hospital of Xiangya Medical College, Central South University, Changsha, China; 8 Department of Neurosurgery, the People’s Hospital of Hainan Province, Hainan, China

**Keywords:** Extracellular matrix, Prognostic markers, CNS cancer

## Abstract

Runt-Related Transcription Factor 1 (RUNX1) is highly expressed in the Mesenchymal (Mes) subtype of glioblastoma (GBM). However, the specific molecular mechanism of RUNX1 in Mes GBM remains largely elusive. In this study, cell and tumor tissue typing were performed by RNA-sequencing. Co-immunoprecipitation (co-IP) and immunofluorescence (IF) were employed to identify members of the RUNX1 transcriptional protein complex. Bioinformatics analysis, chromatin immunoprecipitation (ChIP), and luciferase reporter experiments were utilized to verify target genes. Analyses of The Cancer Genome Atlas (TCGA) and Chinese Glioma Genome Atlas (CGGA) verified the expression levels and prognoses associated with RUNX1/p-SMAD3/SUV39H1 target genes. In vivo patient-derived xenograft (PDX) studies and in vitro functional studies verified the impact of RUNX1 on the occurrence and development of GBM. The results showed that RUNX1 was upregulated in Mes GBM cell lines, tissues and patients and promoted proliferation and invasion in GBM in a TGFβ pathway-dependent manner in vivo and in vitro. We found and verified that BCL3 and MGP are transcriptionally activated by p-SMAD3 /RUNX1, while MXI1 is transcriptionally suppressed by the RUNX1/SUV39H1-H3K9me3 axis. This finding offers a theoretical rationale for using molecular markers and choosing therapeutic targets for the Mes type of GBM.

## Introduction

Glioblastoma (GBM) is known as the most common and malignant form of brain tumors and exhibits heterogeneity in both its morphology and genetics. At present, the standard treatment for GBM is extensive surgical resection, followed by adjuvant radiotherapy and chemotherapy. However, most GBMs will recur in a short time and become resistant to the treatment due to tumor heterogeneity^1–3^. Bulk adult GBM samples have been used to categorize GBM into several distinct subtypes based on global transcription profiles and DNA methylation analyses: proneural, classical or proliferative, and Mes^4–6^. Researchers have found that the standard treatment regimen induces conversion in the tumor expression profile from proneural to Mes^7,8^. To improve clinical outcomes, there is an urgent need for studies aimed at identifying the molecular determinants that govern Mes GBM progression and novel therapeutic targets that can prevent progression.

The transforming growth factor-β (TGFβ) signaling pathway has been associated with a variety of biological contexts including proliferation, epithelial to mesenchymal transition (EMT), and apoptosis^9^. Previous studies have provided both clinical and in vitro evidences showing that the activated TGFβ signaling pathway drives tumor growth. For example, the ligands and receptors associated with the TGFβ signature are present at abnormally high levels in the Mes tumor microenvironment and glioma stem cells (GSCs)^6,10^. SMADs are crucial intracellular nuclear effectors of TGFβ family members. The ligand-induced activation of TGFβ family receptors with intrinsic serine/threonine kinase activity triggers the phosphorylation of receptor-regulated SMADs (R-SMADs), whereas SMAD2 and SMAD3 are phosphorylated by TGFβ and translocated into nucleus^11,12^. Human studies have demonstrated that TGFβ and p-SMAD3 are overexpressed in GBM tissues but undetectable in normal brain tissues, further suggesting that TGFβ contributes to GBM development^13^.

Runt-related transcription factor 1 (RUNX1), also designated AML1, regulates the differentiation of hematopoietic stem cells into mature blood cells^14^. Chromosomal translocations involving the RUNX1 gene are associated with several types of leukemia, including the M2 subtype of acute myeloid leukemia (AML)^15,16^. In central nervous system tumors, RUNX1 has been linked to the Mes state of GBM, in which it maintains the tumor initiating capacity and the ability of tumor cells to invade into the normal tissue. Research on a context-specific regulatory network showed that RUNX1 controls the expression of the Mes signature and is associated with a poor prognosis in GBM^17^. Biochemical analyses confirmed that RUNX1 regulates established drivers of tumor initiation and the Mes subtype via microRNA (miR)-mediated interactions^18^. Moreover, RUNX1 expression is associated with microglial proliferation and activation, and it activates the neuronal differentiation of dorsal root ganglion cell subpopulation^19,20^. RUNX1, RUNX2, and RUNX3, the Runt family members, possess subnuclear targeting signal and SMAD interaction domain^21^. However, the functional roles of RUNX1 in Mes progression have not been fully characterized.

In this study, we first showed that RUNX1 serves as a master regulator in Mes GBM. As a transcriptional regulator, RUNX1 promotes the malignant progression of Mes GBM by significantly affecting the expression of oncogenes and tumor suppressor genes in a TGFβ pathway-dependent manner. These results suggest potential targeted treatment strategies.

## Results

### Upregulated expression level of RUNX1 is displayed in mesenchymal GBM and correlated with poor prognosis

**Fig. 1:**
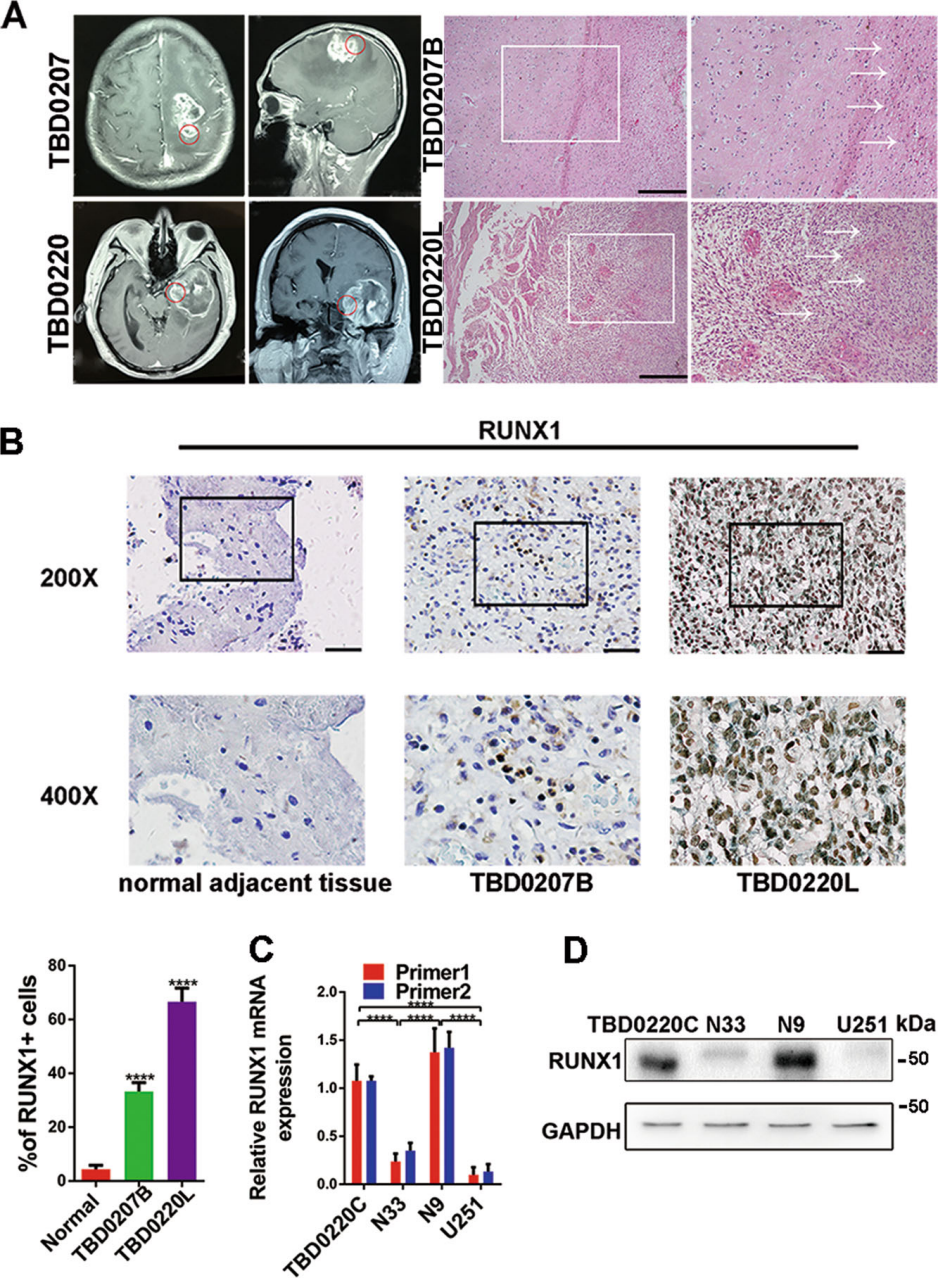
**RUNX1 is upregulated in mesenchymal glioblastoma specimens and cell lines.** **a** TBD0207B and TBD0220L represent samples that were removed from the back and left side of the tumor, respectively. Representative images of HE-stained tissues in TBD0207B and TBD0220L. Scale bar, 100 μm. **b** Representative tissues immunohistochemically stained for RUNX1 in normal brain tissue, TBD0207B and TBD0220L. Scale bar, 100 μm. Quantitative analysis of immunohistochemical staining for RUNX1 in a high-magnification view. *n* = 5 per group. **c**, **d** Western blots and qPCR were used to analyze the protein and mRNA expression levels, respectively, in TBD0220C, N33, N9, and U251 cells. GAPDH served as the negative control. (****p < 0.0001).

It is well known that gene mutation and heterogeneity are existed in most tumors. To investigate whether the RUNX1 has mutation in tumor tissues, we examined TCGA pancancer genomic alterations and found no RUNX1 mutations in the investigated gliomas (Fig. S1). Then, the surgically removed tumor specimens which were cut into six parts: upper, lower, left, right, anterior and posterior were chosen to explore the heterogeneity of gliomas. Compared to the tumor of internal, the external tumor have more glial cells with normal characteristics, so each part of specimens contains the inner and outer parts of the tumor. To further consider GBM heterogeneity, we selected tumor tissues obtained from two patients for histological analysis and transcriptome sequencing^22^, which included front, back, left, and top tumors samples from patient TBD0207 and front, left, and top tumor samples from patient TBD0220. Compared with TBD0207B (the back side of the tumor), TBD0220L (the left side of the tumor) exhibited higher infiltration of tumor cells into normal tissues (Fig. 1a) and higher expression of the invasive protein MMP9 (Fig. S2) by the HE and immunohistochemistry (IHC) staining. The morphological results indicated that TBD0220L exhibited more pronounced invasive characteristics of Mes type^23^. Next, we tested the molecular types of the samples by RNA-seq and found that TBD0220L expressed higher levels of Mes molecular markers, while TBD0207B expressed higher levels of proneural molecular markers (Table S1). We cultured TBD0220L tumor tissue to obtain a stably passaged GBM cell line (TBD0220C) and then performed sequencing of four cell lines (TBD0220C, N9, N33, and U251). The results showed that the TBD0220L and N9 cell lines exhibited Mes cell type characteristics, U251 cells exhibited characteristics of the proneural subtype, and N33 was most similar to the classical subtype (Fig. S3a). Gene set enrichment analysis (GSEA) was used to analyze the molecular phenotype of tissues and cells based on sequencing, which showed that TBD0220L, TBD0220C and N9 displayed a Verhaak Mes phenotype, while TBD0207B and U251 displayed a Verhaak Proneural subtype, which was consistent with the results of RNA sequencing (Figs. S3b and S4a). We selected the marker genes of Mes and Proneural subtype for standardization, and then found that, in our samples and cell lines, the marker genes have higher or closer expression than the samples that have been typed by TCGA (Fig. S4b). Meanwhile, we performed a correlation analysis of Verhaak typing molecular marker genes and found that TBD0220L, TBD0220C, and N9 showed significant positive correlation with each other and significant negative correlation with U251, N33, and TBD0207B (Fig. S5).

Next, immunohistochemical staining (IHC) revealed that the expression of RUNX1 gradually increased from normal brain tissues to TBD0207B and to TBD0220L, and that RUNX1 was mainly expressed in the nucleus (Fig. 1b). Furthermore, the N9 and TBD0220C cell lines expressed higher level of RUNX1 than the N33 and U251 cell lines (Fig. 1c, d). Therefore, RUNX1 expression was highly correlated with Mes GBM.

The proneural, neural, classical, and Mes subtypes were described by a robust gene expression-based molecular classification of GBM. Therefore, we obtained 613 GBM samples (primary and secondary) from patients in the CGGA and TCGA cohorts. The expression level of RUNX1 mRNA was significantly higher in the Mes subtype than in other subtypes (Fig. S6a, b). The receiver operating characteristic (ROC) curve for RUNX1 that separated Mes GBM patients from other patients exhibited high sensitivity in the CGGA and TCGA databases (Fig. S6c, d). Furthermore, when we ranked each specimen from low to high based on the RUNX1 mRNA expression level, GSEA revealed that RUNX1 expression was positively correlated with signatures representative of the Verhaak the Mes subtype (Fig. S6e, f). A Kaplan–Meier survival curve analysis showed an adverse survival in patients with elevated RUNX1 levels (Fig. S7). High level of RUNX1 expression was positively correlated with increased malignancy (WHO grade) (Fig. S8a). In addition, we analyzed the RUNX1 family members RUNX2 and RUNX3, which were also the markers of malignancy, but we found that they were not associated with the Mes subtype and are less sensitive in distinguishing this subtype (Fig. S8b–d).

### Activation of TGFβ pathway strengthened the interaction of RUNX1, p-SMAD3, SUV39H1, and promoted them to translocate into the nucleus

**Fig. 2:**
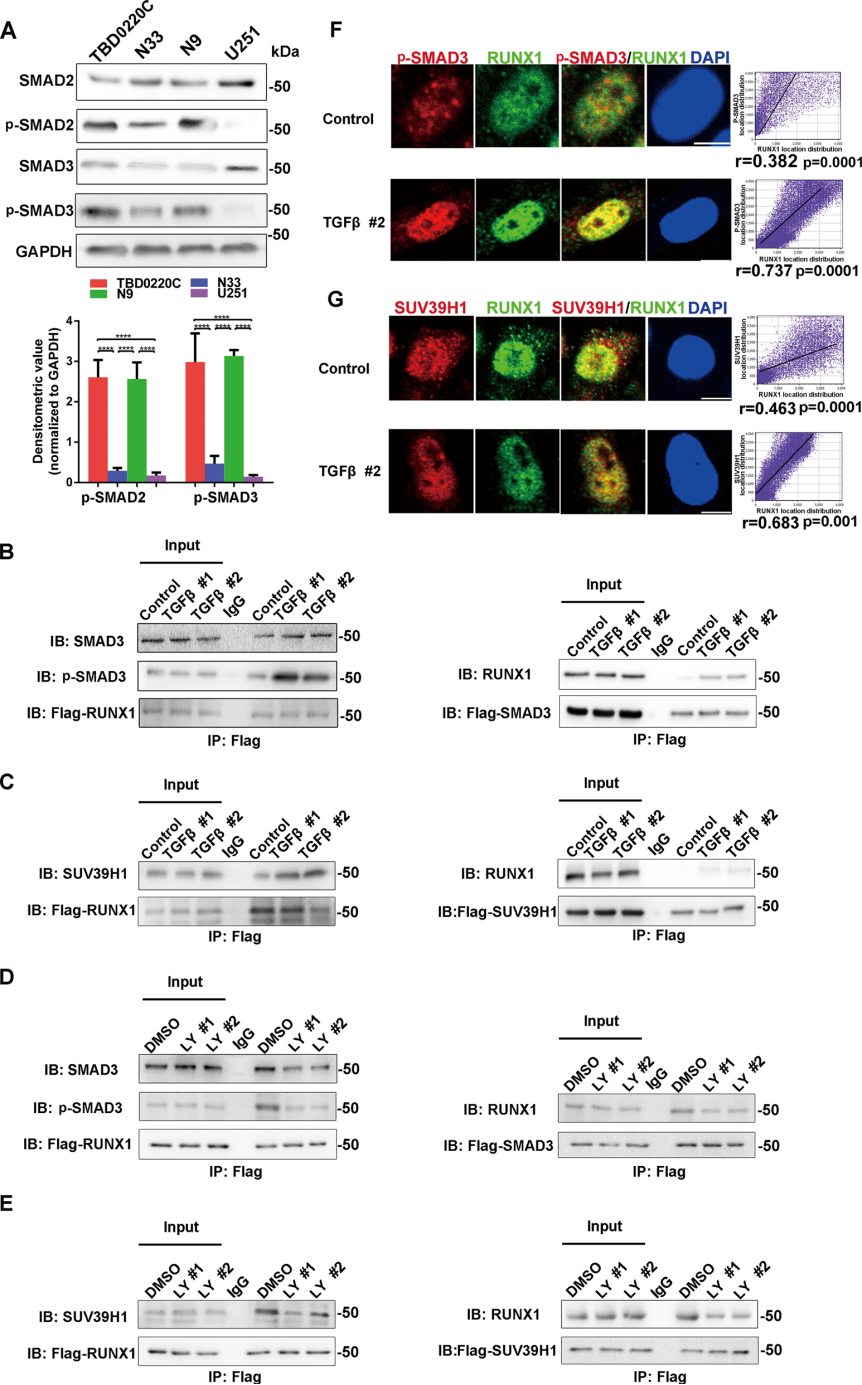
**The TGFβ signaling pathway enhances the protein interaction between RUNX1 and p-SMAD3/ SUV39H1. a** Western blotting analysis of SMAD2, p-SMAD2, SMAD3, and p-SMAD3 expression in four human glioma cell lines. Densitometric analysis of p-SMAD2 and p-SMAD3 is shown. *n* = 3 per group. **b–e** The interaction between RUNX1 and SUV39H1/SMAD3 was validated by co-IP. U251 cells overexpressing Flag-RUNX1 or Flag-SMAD3 (**b**) or Flag-SUV39H1 (**c**) in a background of TGFβ stimulation. N9 cells overexpressing Flag-RUNX1 or Flag-SMAD3 (**d**) or Flag-SUV39H1 (**e**) in the presence of LY2109761. **f**, **g** Subcellular localization of RUNX1, p-SMAD3 (**f**) and SUV39H1 (**g**) in U251 cells under normal or TGFβ protein conditions. Scale bar, 5 μm. A correlation analysis verified the positional overlap. *n* = 5 per group. (TGFβ #1 and TGFβ #2 indicate treatment of TGFβ protein for 4 h and 8 h. LY #1 and LY #2 indicate treatment of LY2109761 for 24 h and 48 h. ****p < 0.0001).

TGFβ signaling pathway was found to be indispensable in Mes tumor development. To investigate the molecular functions of TGFβ signaling pathway in GBM, western blotting was performed to analyze the phosphorylation status of SMAD2 and SMAD3 in the TBD0220C, N33, N9, and U251 cell lines. We found that the Mes cell lines (TBD0220C and N9) presented a high degree of TGFβ pathway activation (Fig. 2a). Using a string protein-protein network, we found five proteins (SMAD3, SMAD4, SUV39H1, CBFβ, and HDAC1) that could be associated with RUNX1, and then co-immunoprecipitation (co-IP) assay confirmed the binding relationships in vivo (Fig. S9a). To further verify the relationship between TGFβ pathway and RUNX1, co-IP experiments were performed to explore the changes in protein binding capacities by establishing different time points to agitate or inhibit the TGFβ pathway via TGFβ protein (activator of TGFβ pathway) or LY2109761 (inhibitor of TGFβ pathway) in U251 and N9 cell lines. By precipitating the corresponding protein with a specific antibody, we found that the activation of TGFβ pathway could facilitate the recruitment of the p-SMAD3/SMAD3 and SUV39H1 proteins by RUNX1 (Fig. 2b, c) and inhibition of TGFβ pathway could weaken the interactions. (Fig. 2d, e). IF via confocal microscopy also revealed that the activated TGFβ pathway induces these three proteins (RUNX1, p-SMAD3, and SUV39H1) to additional sub-cellular location in the nucleus (Fig. 2f, g) (Fig. S9b). In addition, from a whole-cell point of view, the activation of the TGFβ pathway promoted the nuclear translocation of RUNX1 and SUV39H1 (Fig. S9c). Co-IP experiments and Immunofluorescence revealed that the p-SMAD3 and SUV39H1 proteins also bind with each other (Fig. S9d).

### High-throughput sequencing of a GBM cohort showed that BCL3, COL3A1, MGP, POSTN, and MXI1 are potential target genes of RUNX1 and p-SMAD3/SUV39H1

**Fig. 3:**
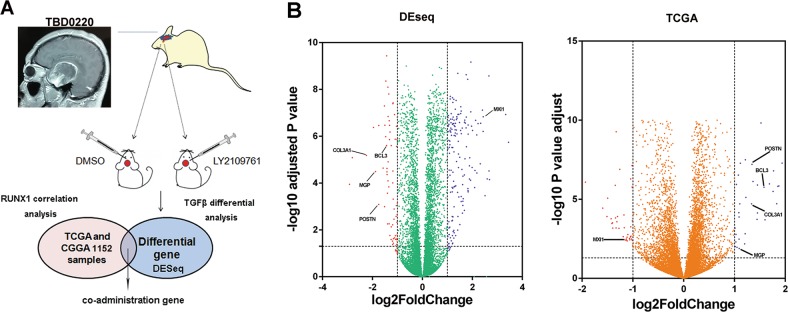
**A bioinformatics analysis revealed a pattern showing that RUNX1-associated gene expression is specifically driven by TGFβ in Mes glioblastoma.** **a** Schematic of the bioinformatics analysis. **b** Volcano plots of the genes that were differentially expressed (red and blue dots) in PDX sequencing data and TCGA datasets. The overlapping genes (BCL3, COL3A1, MGP, POSTN, and MXI1) are marked.

To further explore target genes of RUNX1, the patient-derived tumor xenograft (PDX) GBM tissues obtained from TBD0220L were transplanted into the brains of nude mice. We then treated these mice with DMSO or LY2109761 for 15 days and removed the tumors for RNA sequencing. Among 16,749 differentially expressed genes (DEGs), 1407 were significantly differentially expressed between the control and LY2109761-treated groups, including 574 upregulated and 833 downregulated genes (*p*-value of 0.05 and log fold change (log FC) of 1). RUNX1-related genes were identified in the TCGA and CGGA datasets. RUNX1 and p-SMAD3 are key genes that promote tumor progression and EMT^24,25^. However, RUNX1 associated with SUV39H1 represses transcription by increasing H3K9me3 binding^26^. To identify RUNX1/p-SMAD3-targeted genes, we searched for overlap in genes that were both positively associated with RUNX1 and downregulated by LY2109761. Conversely, RUNX1/SUV39H1-targeted genes were defined as genes that were negatively associated with RUNX1 and upregulated by LY2109761 (Fig. 3a). Clustering patterns were used to present the top 42 encoding genes that were differentially expressed between the DMSO- and LY2109761-treated mice and the top 200 genes that were significantly correlated with RUNX1 expression (Fig. S10a, b). Volcano plots illustrate the distinct transcriptional profiles of genes that were differentially expressed in these two groups. The overlapping genes (BCL3, COL3A1, MGP, POSTN, and MXI1) are shown in Fig. 3b. We performed a correlation analysis of these genes and found that some genes (RUNX1, BCL3, COL3A1, MGP, and POSTN) were positively correlated with each other, and all of these genes were negatively correlated with MXI1 (in contrast to the results for MYC) (Fig. S11a, b) in Mes patients specimens. In addition, in the TCGA and CGGA datasets, the mRNA expression levels of BCL3, COL3A1, MGP, and POSTN were significantly higher in the Mes subtype than in the other subtypes, while MXI1 (in contrast to the results for MYC) was expressed at lower levels in the Mes subtype (Fig. S12).

We obtained Kaplan–Meier survival curves for the TCGA and CGGA data and found that high levels of BCL3, COL3A1, MGP, and POSTN led to a worse prognosis, while a higher level of MXI1 was associated with a better prognosis (Fig. S13a–e).

Functional annotation was performed using GO enrichment analysis to study the biological roles, and the top 200 genes that positively correlated with RUNX1 were analyzed. The most significantly enriched processes associated with the genes were cell adhesion, extracellular matrix organization, and extracellular matrix (ECM)-receptor interactions (Table S2).

### RUNX1 and p-SMAD3 increased the expression of BCL3 and MGP under the TGFβ pathway-activated condition

**Fig. 4:**
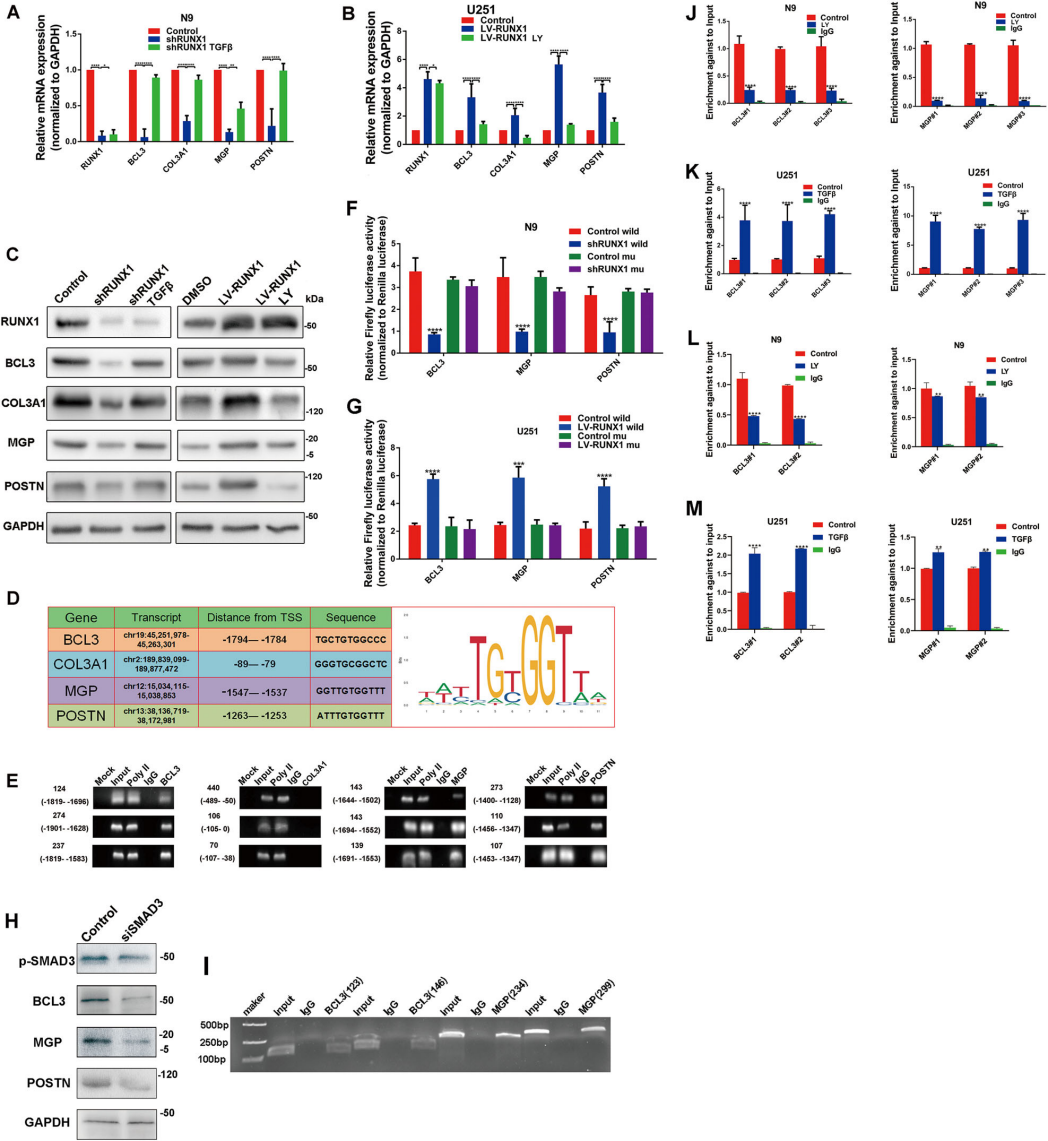
**The TGFβ signaling pathway promotes the expression of BCL3, MGP, and POSTN via the RUNX1/p-SMAD3 transcriptional protein complex.** **a**, **b** Real-time PCR results showing the levels of the RUNX1, BCL3, COL3A1, MGP, and POSTN mRNA after treatment with shRUNX1, shRUNX1 plus TGFβ protein, LV-RUNX1 or LV-RUNX1 plus LY2109761 treatment in U251 cells or N9 cells. **c** Western blots were performed to analyze the corresponding protein expression level. GAPDH was used as a loading control. **d** A table showing the distance from the transcript start site and the binding sequence for BCL3, COL3A1, MGP, and POSTN (including the RUNX1 binding sequence). **e** ChIP analysis of the ability of RUNX1 to bind to the BCL3, COL3A1, MGP, or POSTN promoter using antibodies against RUNX1 in 293T cells. **f** Luciferase activity is shown for the control reporter, the wild-type reporter and the mutant reporter with the control or shRUNX1 in N9 cells. **g** Luciferase activity is shown for the control reporter, the wild-type reporter and the mutant reporter with the control or LV-RUNX1 in U251 cell line. **h** Western blots were performed to analyze the protein expression of p-SMAD3, BCL3, MGP, and POSTN with the treatment of control or siSMAD3. **i** ChIP analysis of the ability of p-SMAD3 to bind to the BCL3 or MGP promoter (or coding region) using antibodies against p-SMAD3 in 293T cells. **j**, **k** ChIP-PCR analyses showing the changes that occurred in the ability of RUNX1 to bind the promoters of target genes after U251 or N9 cells were treated with TGFβ or LY2109761, respectively. **l**, **m** ChIP-PCR analyses showing the changes that occurred in the ability of p-SMAD3 to bind the promoters of target genes after N9 and U251 cells were treated with LY2109761 or TGFβ, respectively. (*p < 0.05, **p < 0.01, ***p < 0.001 and ****p < 0.0001, respectively).

Next, we investigated the regulation mechanism between RUNX1 and these target genes. RUNX1 overexpression and knockdown lentivirus constructs were designed (LV-RUNX1 and shRUNX1, respectively) and identified by real-time quantitative PCR (qPCR) and western blot assays (Fig. S14a, b). Then, mRNA and protein expression levels of BCL3, COL3A1, MGP, and POSTN were detected by qPCR and western blot assays. As shown in Fig. 4a–c, depletion of RUNX1 decreased the expression of these genes in N9 cells, while the reverse was true when RUNX1 was over-expressed in U251 cells. By PROMO and TransFac program analysis, we found that RUNX1 was a potential transcription factor for their promoters. We assigned a p*-*value based on correspondence between the original sequence ranking, which were based on the experimental binding scores, and found the RUNX1 binding sequence (JASPAR, http://jaspar.binf.ku.dk/) and putative binding sites upstream of the transcriptional start codon (Transcription Factor BINDing site (TFBIND), https://omictools.com/transcription-factor-binding-site-tool) (Fig. 4d). Then we performed a ChIP-PCR assay to study the association between the RUNX1 and promoters of target genes in HEK 293T cells. As shown in Figs. 4e and S15a we found that promoter fragments with RUNX1 binding sites, but not RUNX1-negative genomic regions of these genes (BCL3, MGP, and POSTN, but not COL3A1), were effectively enriched by an anti-RUNX1 antibody. We also employed a Luciferase reporter assay to validate the correctness of the targeting sequence. We cloned and inserted a wild type or mutant binding sequence into a luciferase reporter vector-PGL4.1 (Table S3). The results demonstrated that in U251 cells, overexpression of RUNX1 markedly enhanced the luciferase activity of the wild-type reporter, but not the mutant reporter, and shRUNX1 treatment resulted in a lower level of luciferase activity in N9 cells with the wild-type reporter, but not with the mutant reporter (Fig. 4f, g). Since these target genes were regulated by RUNX1 and TGFβ pathway, we treated the RUNX1-knockdowned N9 cells with TGFβ protein and RUNX1-overexpressed U251 cells with LY2109761. We found that decreased changes of these genes were rescued via TGFβ protein treatment, and vice versa (Fig. 4a–c). To further investigate the mechanism, we knockdowned SMAD3 (the downstream gene of TGFβ) by siRNA and found that the expressions of p-SMAD3, BCL3, MGP, and POSTN were reduced (Fig. 4h). We searched putative binding sites of p-SMAD3 by ENCODE TF dataset in UCSC (https://genome.ucsc.edu), and found that promoters of BCL3 and MGP have the binding motif. ChIP-PCR assay confirmed that target sequences of BCL3 and MGP were efficiently enriched by an anti-p-SMAD3 antibody (Fig. 4i).

Furthermore, inhibition of TGFβ pathway in N9 cells reduced the enrichment of RUNX1 and p-SMAD3 at the promoter regions of these two genes, while activation of TGFβ pathway increased the enrichment of DNA fragments in the U251 cell line (Fig. 4j–m). To detect whether the protein binding sites of p-SMAD3 and RUNX1 are the DNA binding regions, we mutated their DNA binding domains. IP experiments confirmed that they still bind to each other, indicating that the binding regions of these two protein are respective non-DNA binding domains (Fig. S15b). It was also demonstrated that their binding does not affect the transcriptional regulation of DNA.

These results above manifested that BCL3 and MGP were regulated by RUNX1 and p-SMAD3 in a TGFβ pathway-dependent manner.

### TGFβ signaling decreased the expression of MXI1 via RUNX1/SUV39H1-mediated H3K9me3 modifications, and regulated the expression of target genes (BCL3, MGP, and MXI1)

**Fig. 5:**
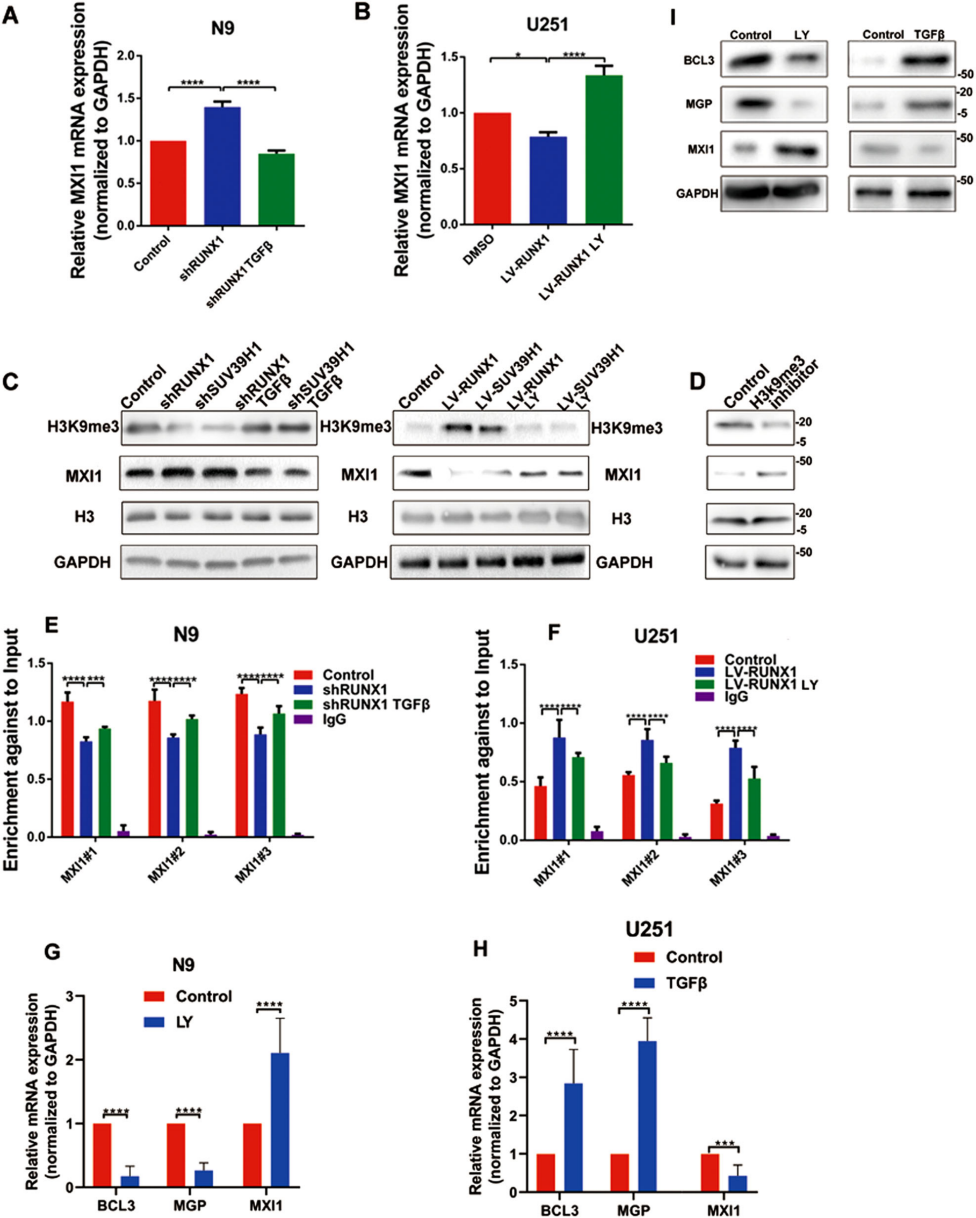
**The TGFβ signaling reduces the expression of MXI1 via the RUNX1/ SUV39H1 transcriptional complex, and regulates the expression of target genes (BCL3, MGP, and MXI1).** **a**, **b** Real-time PCR showing the effect of shRUNX1, shRUNX1 plus TGFβ, LV-RUNX1, or LV-RUNX1 plus LY2109761 on MXI1 mRNA levels in U251 or N9 cells. **c** Western blots were performed to analyze the protein expression of MXI1 and H3K9me3 after treatment with shRUNX1/LV-RUNX1 or shSUV39H1/LV-SUV39H1 or rescue with TGFβ/LY2109761. GAPDH and H3 were used as loading controls. **d** Western blots were performed to analyze the protein expression of MXI1 and H3K9me3 after treatment with H3K9me3 inhibitor. GAPDH and H3 were used as loading controls. **e** ChIP-qPCR results showing the level of H3K9me3 on the MXI1 promoter after treatment with shRUNX1 or shRUNX1 plus TGFβ. **f** The level of H3K9me3 enrichment on the MXI1 promoter after LV-RUNX1 or LV-RUNX1 plus LY2109761. **g**, **h** Real-time PCR showing the effect of LY2109761 or TGFβ on BCL3, MGP, POSTN, and MXI1 mRNA levels in U251 or N9 cells. **i** Western blots showing the effect of LY2109761 or TGFβ on BCL3, MGP and MXI1 protein levels in U251 or N9 cells. (*p < 0.05, ***p < 0.001 and ****p < 0.0001, respectively).

As shown in Fig. 3b, expression of RUNX1 was negative correlated with MXI1. SUV39H1 is a histone methyltransferase which is combined with RUNX1 (Fig. 2c, e). QPCR and western blot assays showed that decreased RUNX1 and SUV39H1 could increased the expression of MXI1 and the adding TGFβ protein reversed this effect (Fig. 5a, c). Opposite results were obtained in U251 cells with overexpression of RUNX1 and SUV39H1 or LY2109761 (Fig. 5b, c). H3K9me3 is implicated in transcriptional silencing. Depletion of RUNX1 and SUV39H1decreased tri-methylation of H3K9 and TGFβ protein treatment could rescue the level of H3K9me3 (Fig. 5c). We utilized H3K9me3 inhibitor to treat cells and found downregulation of H3K9me3 and upregulation of MXI1 (Fig. 5d). Therefore, to determine the functional role of H3K9me3 at the MXI1 promoter, we performed a ChIP assay coupled with qPCR analysis to detect specific binding. We analyzed the MXI1 promoter region within the distal promoter in GSE103408 (H3K9me3 ChIP-seq in the GBM U87 cell line). The results showed that H3K9me3 was enriched in the promoter region of MXI1 (Fig. S16). In N9 cells, downregulation of RUNX1 decreased H3K9me3 binding to the MXI1 promoter, and the TGFβ protein reversed this inhibitory effect. Furthermore, we observed the same phenomenon when U251 cells were treated with LV-RUNX1 and LY2109761 (Fig. 5e, f). These results suggested that RUNX1/SUV39H1 interacts with H3K9me3 via the TGFβ pathway leading to the silencing of MXI1. Naturally, the TGFβ pathway directly affected the expression of target genes (BCL3, MGP, and MXI1) (Fig. 5g–i).

### RUNX1 promoted cell proliferation, invasion, and adhesion in a TGFβ pathway-dependent manner in vitro

**Fig. 6:**
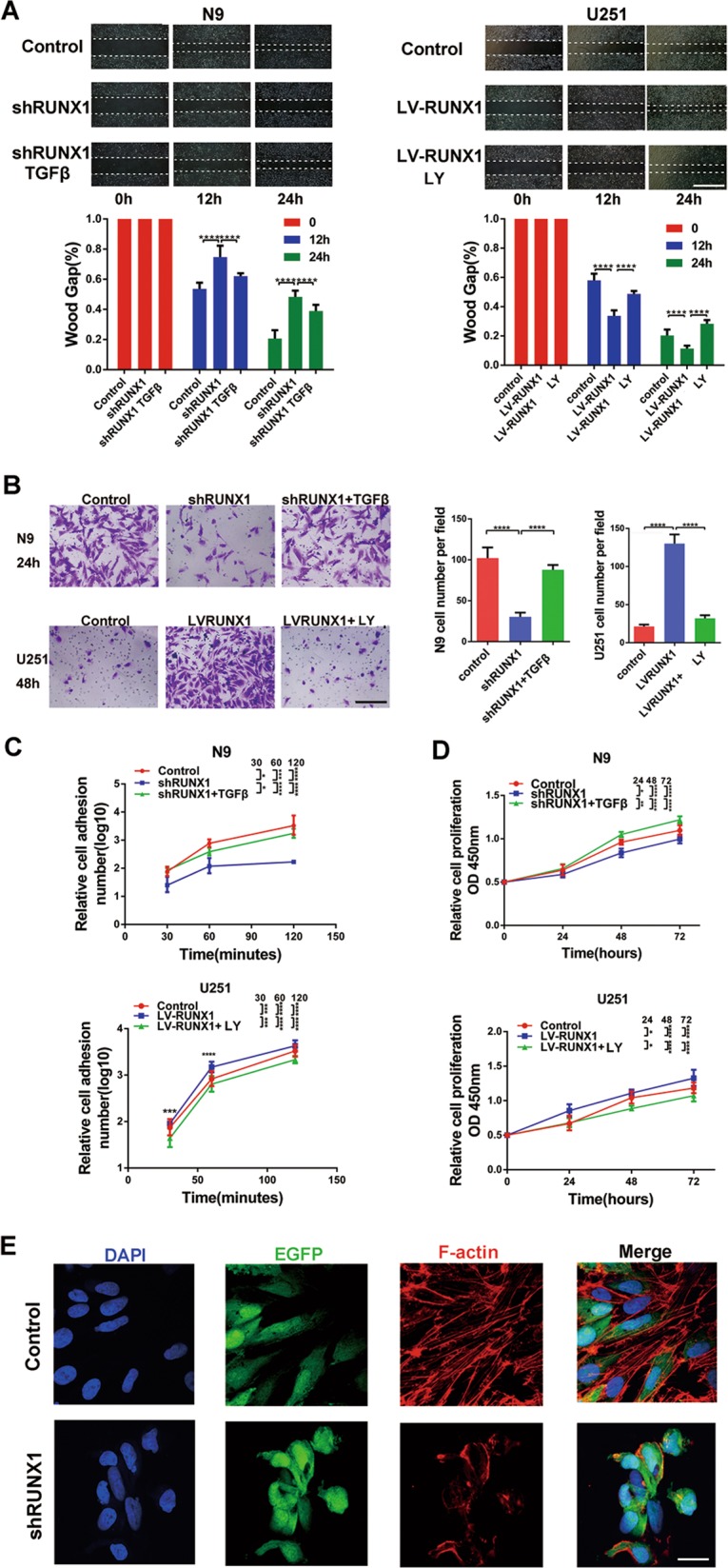
**RUNX1 promotes cell proliferation, invasion and adhesion in a TGFβ pathway-dependent manner in vitro.** **a** Wound-healing assays were used to analyze migration and invasion following treatment with shRUNX1, TGFβ plus shRUNX1, LV-RUNX1, or LY219761 plus LV-RUNX1. Scale bar, 200 μm. *n* = 3 per group. **b** N9 cells were treated with shRUNX1 or TGFβ plus shRUNX1, and U251 cells were treated with LV-RUNX1 or LY219761 plus LV-RUNX1, and Transwell assays were performed. Scale bar, 60 μm. *n* = 3 per group. **c**, **d** CCK8 assays and cell adhesion assays were used to analyze the proliferation and adhesion, respectively, of GBM cells. **e** F-actin levels were analyzed by immunofluorescence in control and shRUNX1-treated N9 cells that were transfected with EGFP lentivirus. Scale bar, 20 μm. (*p < 0.05, ***p < 0.001, and ****p < 0.0001, respectively).

To clarify the function of RUNX1 in vitro, shRUNX1 or LV-RUNX1 were respectively transfected into N9 or U251 GBM cells to evaluate their changes and verify whether their effects could be reversed by the TGFβ protein or LY2109761. We investigated the effects on motility in wound-healing and Transwell assays. The results suggested that RUNX1 increased migration and invasion in a TGFβ pathway-dependent manner (Fig. 6a, b). To further explore the function of RUNX1, CCK8 assays and cell adhesion assays were performed, and the results showed that, overexpression of RUNX1 and activation of TGFβ pathway enhanced proliferation and adhesion in GBM cells (Fig. 6c, d). Immunofluorescence results showed that knocking down RUNX1 caused the F-actin lose in N9 cells (Fig. 6e).

### RUNX1 promoted proliferation and invasion in PDX GBM in a TGFβ pathway-dependent manner in vivo

**Fig. 7:**
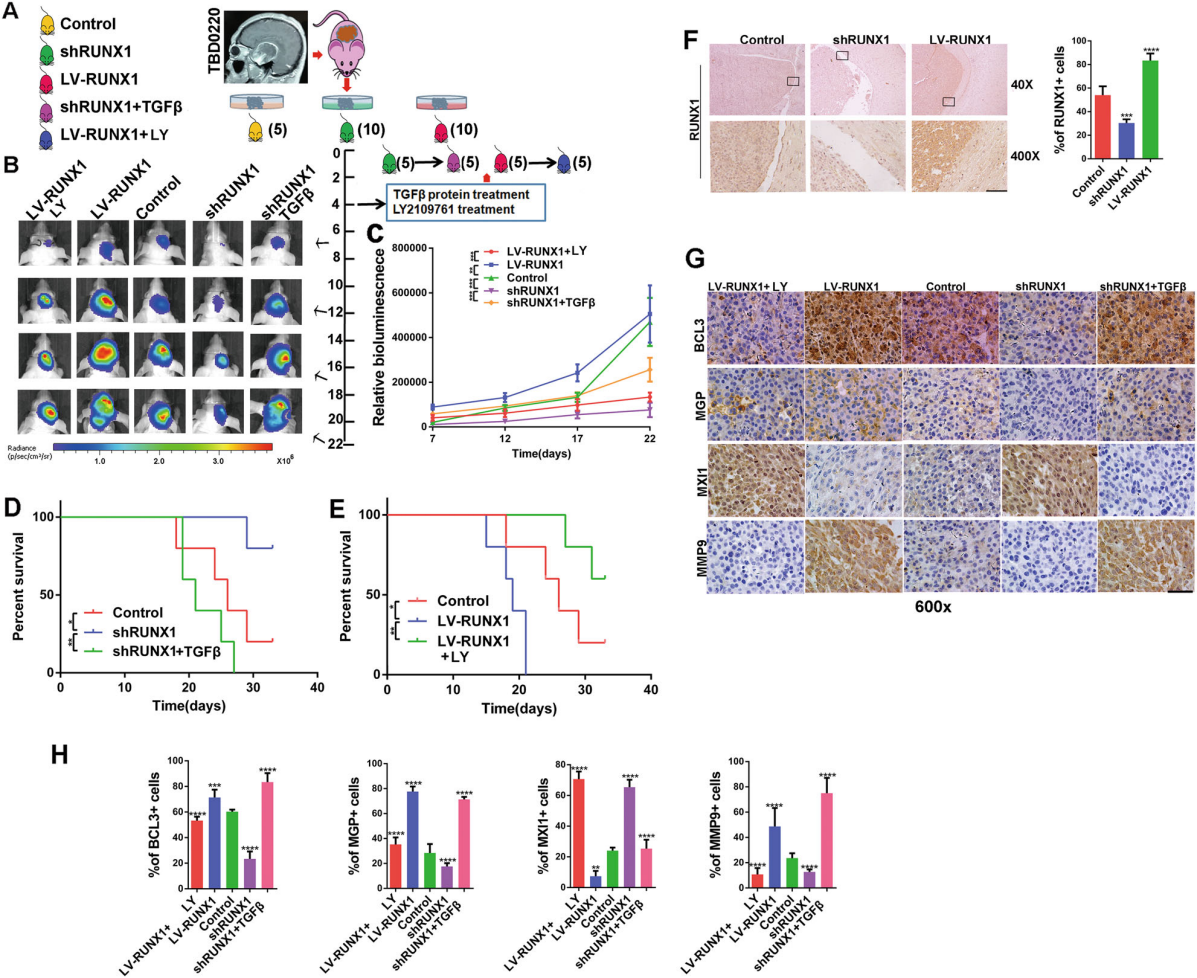
**RUNX1 promotes PDX tumor proliferation and invasion in a TGFβ pathway-dependent manner in vivo. a** First, nude mice were intracranially implanted with differently treated PDX tissues (control, shRUNX1, and LV-RUNX1). After 4 days LY2109761 and TGFβ were administered via the tail vein, the nude mice were divided into 5 groups, and bioluminescence imaging was performed. **b** Representative bioluminescence images of mice implanted with intracranial tumors on days 7, 12, 17 and 22. The relative bioluminescence values are shown (**c**). Survival was also measured in these 5 groups (**d**, **e**). (*, ** and *** indicate p < 0.05, p < 0.01and p < 0.001, respectively). **f** IHC was used to analyze the expression of RUNX1 at the control, shRUNX1 and LVRUNX1 sites.Quantitative analyses were performed using ImageJ software for each high-magnification view. n = 5 per group. **g** IHC staining for BCL3, MGP, MXI1 and MMP9 was performed in mouse brain slices in the control, shRUNX1, TGFβ protein plus shRUNX1, LV-RUNX1 and LY2109761 plus LV-RUNX1 groups. **h** Quantitative analyses of these genes were performed using ImageJ software for each high-magnification view. *n* = 5 per group. Scale, 20 μm. (**p < 0.01, ***p < 0.001 and ****p < 0.0001, respectively).

We used a mouse PDX model (TBD0220L-derived) to verify our previous findings in vivo (Fig. 7a). We evaluated the effects of the RUNX1 and TGFβ/RUNX1 axis on tumor growth, and detected the tumor growth by bioluminescence analysis. Compared with the control-treated tumors, the shRUNX1-treated tumor volumes were significantly smaller. The LV-RUNX1-treated mice exhibited the opposite effects. The proliferation ability of tumor was enhanced by TGFβ activation, while was inhibited by TGFβ inactivation (Fig. 7b, c). We found that both TGFβ and RUNX1 were poor prognostic factors via the survival analysis of nude mice (Fig. 7d, e).

IHC analysis of RUNX1 and p-SMAD3 confirmed that viral transfection was efficient and the TGFβ pathway was regulated (Fig. 7f) (Fig. S17a). IHC was used to compare the expression levels of target genes (BCL3, MGP, and MXI1) in the 5 groups. The expression levels of BCL3 and MGP were decreased by the shRUNX1 lentivirus and rescued by the addition of TGFβ protein, while the expression level of MXI1 was decreased by the LV-RUNX1 lentivirus and this effect was reversed by LY2109761 (Fig. 7g, h). To predict tumor invasiveness, we detected the expression of MMP9 in corresponding tumor sections. The invasive ability of Mes GBM was weakened by shRUNX1 lentivirus treatment and rescued by TGFβ protein treatment (Fig. 7g, h). The invasive ability of Mes GBM was also directly weakened by LY2109761 (Fig. S17b).

## Discussion

GBM is the most malignant type of glioma (WHO grade IV). After diagnosis, despite the use of aggressive surgery, radiation, and chemotherapies, the average lifetime is 14 months^27^. Of the four molecular subtypes (classical, Mes, neural, and proneural), the Mes phenotype is associated with the highest degree of aggressiveness and treatment resistance and therefore presents the worst survival rate. The worse point was that the researchers found multitherapy-resistant glioma cells have a Mes character^28^. We did not include neural subtype in the classification because this subtype arose from contamination of the original samples with nontumor cells^29^. However, TCGA and CGGA still include this type. This analysis did not affect our experimental process or the reliability of results.

In the hematological and immune systems, AML-1 (RUNX1) is required for the maturation of megakaryocytes and the differentiation of cells including T cells and B cells^30^. We hypothesize that the overexpression of RUNX1 in Mes GBM, especially in cancer stem cells, may cause tumor cells to over differentiate, thereby increasing their ability to attack and spread. By analyzing data in TCGA and CGGA, we found that RUNX1 is much more highly expressed in the Mes type than in other subtypes. These data suggested that RUNX1 may be usable as a molecular marker of Mes GBM. Although RUNX2 and RUNX3 were also the malignant predictors of glioma, they have a poor specificity for distinguishing Mes types (Fig. S8). Our results allowed us to define RUNX1 as a Mes GBM driver. Furthermore, molecular tools for the prevention of cancer can be based on the use of natural or synthetic agents that interrupt the prime drivers or key derangements or the context in which these drivers act or in which the derangements occur^31^. Therefore, we believe that RUNX1 can be used as a candidate target for molecular therapies.

The induction of TGFβ signaling is also a potential signature of the Mes subtype of GBM^32^. The Western blots shown in Fig. 3a demonstrated that Mes cell lines (i.e., TBD0220C and N9 cells) present a high degree of TGFβ pathway activation. Moreover, RUNX1 may work alone or as a complex with other proteins to promote (as an activator) or block (as a repressor) the recruitment of RNA polymerase to specific genes^33,34^. When we combined these two findings in the String protein network, we verified that the interaction of RUNX1 with p-SMAD3/SUV39H1 and the degree of their integration are modulated by TGFβ. P-SMAD3 could also bind directly to the promoter or coding region of BCL3 and MGP to activate their transcription. TGFβ signaling decreased the expression of MXI1 via RUNX1/SUV39H1-mediated H3K9me3 modifications.

Among its target proteins, BCL3 functions as a transcriptional coactivator that activates via its association with NF-κB homodimers^35^. NF-κB pathway activation is integrated into the Mes signaling network and is related to master transcription factors and promotes Mes differentiation in GBM^8^. MGP acts as a migration-promoting Mes component in GBM. As an invasion stimulator and ECM component, MGP underlies the unfavorable prognosis of GBMs with mesenchymal gene expression profiles^36,37^. MXI1 negatively regulates members of the c-Myc family, and c-Myc activates transcription and stimulates cell proliferation^38^. A search and verification of downstream targets revealed that RUNX1 plays a major role in the malignant progression of Mes GBM, affecting the formation of extracellular matrix through MGP and promoting the ability of tumor cells (especially cancer stem cells) to invade into normal tissues. BCL3 protein expression alters the transcriptomic and epigenetic signatures of tumor cells via the NF-κB pathway and changes the tumor microenvironment by inducing the secretion of cytokines. Finally, inhibiting MXI1 accelerates the cell cycle transition by decreasing antagonism with c-Myc.

In summary, this study demonstrates that RUNX1 is highly expressed in Mes GBM and therefore a potential therapeutic target. Some genetic testing methods may be feasible for determining whether tumor tissues highly express RUNX1 and the corresponding downstream genes (BCL3, MGP, and MXI1), consistent with our experimentally validated results: in positive cases, targeting RUNX1 may be a suitable optional treatment. We are now seeking a preclinical study of RUNX1 inhibitors in the near future. Galunisertib, a TGFβ receptor (R) 1 kinase inhibitor, is currently being tested in a phase II randomized trial involving patients with recurrent GBM^39^. A combination therapy consisting of TGF-β pathway inhibitors and RUNX1 inhibitors may also represent a promising prospect.

## Materials and methods

### Samples used for RNA sequencing and microarray data

In total, mRNA microarray data 130 samples were obtained from the Chinese Glioma Genome Atlas (CGGA, http://www.cgcg.org.cn/) dataset and included in this study (Table S4). Additionally, 483 (AgilentG4502A_07_2) and 539 (AffyU133a) GBM samples, including samples with known mutations and mRNA expression levels, were obtained from The Cancer Genome Atlas (TCGA, http://cancergenome.nih.gov/) datasets. The US National Cancer Institute Repository for Molecular Brain Neoplasia Data cohort (REMBRANDT: http://caintegrator.nci.nih.gov/rembrandt/, *n* = 474) was also analyzed.

In 2017, 2 patients (TBD0207 and TBD0220) with GBM and with no history of radiation therapy or chemotherapy underwent surgical treatment at Hebei University Affiliated Hospital (Baoding, China) and allowed their tumor tissues to be collected (Table S5). The primary cell lines used in this study were derived from GBM tissues. TBD0220C was obtained from TBD0220L tissue culture. Tumor tissues treated with or without LY2109761 (a TGFβ/SMAD inhibitor, Selleck) and cell lines (N9, N33, TBD0220C, U251) were sequenced at the Beijing Genomics Institute (BGI, Beijing, China) (Table S1). Complete clinical data were collected, and the collection and processing of primary human GBM tumor samples were performed in accordance with the ethical standards of the 2008 Helsinki Declaration. All patients provided written consent for the use of their samples in biomedical research. Tumor grades were determined according to the 2016 World Health Organization (WHO) classification of nervous system tumors. Hierarchical clustering of differential expression was analyzed by Cluster 3.0. Before a heatmap of the data was constructed, log-transformed data were further centered according to the mean value for the gene (centered by gene) and normalized.

### Cell culture and treatments

The U251 and 293T cell lines were purchased from ATCC (American Type Culture Collection, Manassas, VA, USA) and cultured in Dulbecco’s modified Eagle’s medium (DMEM) supplemented with 10% heat-inactivated fetal bovine serum (FBS, Gibco BRL, Rockville, MA). N9 and N33 patient-derived cells (Table S1) were obtained from Professor Fan at Beijing Normal University (BNU) and grown in DMEM/F12 (1:1; Gibco BRL, Rockville, MA) supplemented with 10% FBS. Cells were grown at 37 °C in 5% CO_2_. All GBM cells except those cultured in vivo cultures were maintained for fewer than eight generations. We treated cells with TGFB1 protein (Sino Biological, Beijing, China) at a final concentration of 10 ng/ml, LY2109761 (Selleck, Shanghai, China) at a final concentration of 2 mg/ml and BIX01294 (Selleck, Shanghai, China) at a final concentration of 50 μM.

### Lentivirus, plasmids, siRNA, and transfection

The shRUNX1 and LV-RUNX1 lentiviruses were obtained from Genechem (Shanghai, China). The siSUV39H1 siRNA was obtained from Genepharma (Shanghai, China). The LV-SUV39H1 plasmid was obtained from (Vigenebio, Jinan, China). The shRUNX1 target sequence, siSMAD3 sequence and siSUV39H1 sequence are provided in Table S6. Flag-RUNX1RHD-del (del 135–167) and Flag-SMAD3MH1-del(del 57–94) were created by polymerase chain reaction with the insertion of a BglII restriction site to join the fragments. The mutated sequence was inserted into the PCMV-N-FLAG series vectors, which was made by IBSBIO(Shanghai, China), based on the website (http://asia.ensembl.org/). Mutated or wild-type promoters containing the putative target regions of BCL3, MGP and POSTN were synthesized and cloned into pGL4.10 [luc2] (Promega, Madison, WI, USA) vector sites and the pGL4.70 [hRluc] (Promega Madison, WI, USA) was used as a promoter-less control vector. The open reading frames (ORFs) of RUNX1, SMAD3, and SUV39H1 were cloned with a C-terminal Flag into pENTER (Vigenebio, Jinan, China). The siRNAs and plasmids were transfected into cells using Lipofectamine 3000 (Invitrogen, USA) according to the manufacturer’s protocol.

### Immunohistochemistry, HE staining, and Quantitative real-time PCR

IHC, HE, and qPCR assays were performed as described in the Supplementary Materials and Methods.

### Western blot analysis and coimmunoprecipitation assay

Proteins were extracted from the cells, and western blotting was completed as described previously^40^. For coIP assay, the cells were lysed using Western and IP lysis buffer (Beyotime Biotechnology, China) and incubated with 40 ml of protein-A/G PLUS agarose beads (Millipore, USA) and 1 mg of antibodies at 4 °C overnight. After the samples were washed three times with RIPA buffer, they were analyzed by western blot analysis.

### Immunofluorescence and confocal imaging

Confocal microscopy was performed as previously described^41^. Confocal images of the cells and correlation analyses of protein localization were acquired with confocal microscope (FV500) using FluoView software (Olympus).

### Wound-healing assay, cell proliferation assay, cell invasion assay, and cell adhesion assay

Wound-healing assays and cell invasion assays were performed as previously described^41^. Cell proliferation and cell adhesion assays were performed as described in the Supplementary Materials and Methods.

### Chromatin immunoprecipitation and dual luciferase reporter assay

Cells were harvested for chromatin immunoprecipitation (ChIP) using an EZ-ChIP Kit (Millipore, USA) according to the manufacturer’s protocol. The chromatin was extracted, and cross-linked DNA was cut into segments of ~200–1000 base pairs. Protein G agarose was added to the antibody/chromatin complexes, and the mixture was incubated overnight at 4 °C. RUNX1 (ab23980, Abcam, UK), p-SMAD3 (#9520, Cell Signaling Technology, USA), and H3K9me3 (#13969, Cell Signaling Technology, USA) antibodies were used to pull down DNA from formaldehyde cross-linked chromatin. The protein G agarose antibody/chromatin complexes were resuspended in wash buffer and centrifuged to collect the protein/DNA complexes. The protein/DNA cross-links were cleaved to yield free DNA. Purified DNA was resuspended in TE buffer for PCR. In addition, some purified DNA was quantified using real-time quantitative PCR.

N9 or U251 cells were cultured in a 12-well plate and cotransfected with pGL4.10 plasmid vectors carrying either wild or mutated sequences together with pGL4.70. Firefly and Renilla luciferase activities were measured 48 h after transfection using a dual-luciferase reporter assay system (Promega, USA). The luciferase activity was calculated as the ratio of firefly luciferase intensity to renilla luciferase intensity.

### Intracranial patient-derived xenograft experiments

All animal experimental protocols were approved by the Tianjin Medical University Animal Care and Use Committee. First, BALB/c-A nude mice (3–4 weeks old) were purchased. Then, we collected 13 intracranial GBM specimens from patients treated at the Affiliated Hospital of Hebei University. The specimens were subcutaneously implanted in nude mice, in which the samples obtained from patient TBD0220L survived and grew. Viable tumor tissues were removed and then triturated in serum-free DMEM/F12 medium. The disrupted tissues were filtered and suspended, and the shRUNX1 and the LV-RUNX1 were added. The mixtures were then incubated for 6 h. Finally, the tissues were stereotactically implanted using cranial guide screws into the intracranial region in nude mice. Bioluminescence imaging was used to detect intracranial tumor growth in the mice on days 7, 12, 17, and 22. In the meantime, the overall mouse survival time was monitored. Beginning the 4th day after transplantation, we injected the mice with LY2109761 (50 mg/kg) and TGFβ proteins (4 mg/kg) via the tail vein once every other day. After death the brains of the mice were carefully extracted and fixed in 10% formalin.

### Statistics

All experiments were analyzed using the mean results from three independent experiments. The chi-squared test was used to determine whether there was a significant difference between two groups. Survival curves were drawn using Kaplan–Meier survival plots, and the log-rank test was used to test significance. Correlations between tissues were evaluated using two-sided Pearson’s correlation tests. Statistical significance was determined using Student’s *t*-test or ANOVA for functional analyses. Pathway and Gene Ontology (GO) analyses were performed using DAVID (http://david.abcc.ncifcrf.gov/). Heat maps were constructed using Gene Cluster 3.0 and Gene Tree View software. Gene Set Enrichment Analysis (GSEA) was performed. Volcano maps were constructed with GraphPad Prism 6. All statistical analyses were performed using SPSS 22.0 software and GraphPad Prism 6. A *p*-value <0.05 was regarded as statistically significant.

## Supplementary information

table s7

Supplementary materials and methods

figure s1

figure s2

figure s3

figure s4

figure s5

figure s6

figure s7

figure s8

figure s9

figure s10

figure s11

figure s12

figure s13

figure s14

figure s15

figure s16

figure s17

Supplementary figure legends

table s2

table s3

table s4

table s5

table s6

Table S1
