# Supplementary material for: RUNX1 contributes to the mesenchymal subtype of glioblastoma in a TGFβ pathway-dependent manner
Source: Cell Death Dis. 2019 Nov 21;10(12):877. doi: 10.1038/s41419-019-2108-x (PMC6872557; doi:10.1038/s41419-019-2108-x)
Supplement: Supplementary file 1 — table s7 [file 41419_2019_2108_MOESM1_ESM.docx]

Table S7.

Primers of PCR

|  | Forward Primer | Reverse Primer | Purpose |
| --- | --- | --- | --- |
| RUNX1#1 | CTGCCCATCGCTTTCAAGGT | GCCGAGTAGTTTTCATCATTGCC | q-PCR |
| RUNX1#2 | TGAGCTGAGAAATGCTACCGC | ACTTCGACCGACAAACCTGAG | q-PCR |
| BCL3 | GCCTACACCCCTATACCCCA | GATGTCGATGACCCTGCGG | q-PCR |
| COL3A1 | AATCAGGTAGACCCGGACGA | CTCCTGGGATGCCATTTGGT | q-PCR |
| MGP | GAGCCTGATCCTTCTTGCCA | GTGGACAGGCTTAGAGCGTT | q-PCR |
| POSTN | CAACGCAGCGCTATTCTGAC | TCGGAAGCCACTTTGTCTCC | q-PCR |
| MXI1 | GGTGCCCCTTCTCAGACATTT | AGTAACCCTCGTCACTCCCA | q-PCR |
| SUV39H1 | AACTATCCACGCTGCTCGAA | GTTCAATCCGCCCCTCATCA | q-PCR |
| GAPDH | AGCCACATCGCTCAGACAC | GCCCAATACGACCAAATCC | q-PCR |
| BCL3#1 | CCAACCGAATTCCTCCCCTC | CTCAGACAAGCCATCCCCTG | ChIP-qPCR |
| BCL3#2 | ATAGTGCTCCACACCCCTCT | TTCATGGCGAAACATCCCGA | ChIP-qPCR |
| BCL3#3 | CCAACCGAATTCCTCCCCTC | CCCTTCCCTTCCTTTCGACC | ChIP-qPCR |
| COL3A1#1 | GGTGGCATTTCTTTCCGTGAG | CACTTTCCAGCCCCTTTCTGA | ChIP-qPCR |
| COL3A1#2 | TTTACTGCTGAGGGGATGGG | ACTGAGATTCCTTTGCTTTGGC | ChIP-qPCR |
| COL3A1#3 | CTTTTACTGCTGAGGGGATGG | TTTGGCTTCCCTCACTTTCCA | ChIP-qPCR |
| MGP#1 | TAGTGAACTGGCTTTCGCACT | TCCACTAATGAGACTGGCGG | ChIP-qPCR |
| MGP#2 | TAGTGAACTGGCTTTCGCACT | TCCACTAATGAGACTGGCGG | ChIP-qPCR |
| MGP#3 | TGAACTGGCTTTCGCACTTT | CCACTAATGAGACTGGCGGTT | ChIP-qPCR |
| POSTN#1 | CACAAAGGACCAGGGTCAGAA | GGCTTTAGGGGAACTGGACT | ChIP-qPCR |
| POSTN#2 | GCTCTTCTTTTGAATTGCCCC | GAAAGCACACAGTGAAACCACA | ChIP-qPCR |
| POSTN#3 | GCTCTTCTTTTGAATTGCCCCTT | AGCACACAGTGAAACCACAAA | ChIP-qPCR |
| MXI1#1 | TCCCATTTGGATTGGGGAGC | CTCTGGGTGCACCTGCATAA | ChIP-qPCR |
| MXI1#2 | AGCAGCCTTCAATCATCCCC | GTTTCACTGCCACTGCTGTC | ChIP-qPCR |
| MXI1#3 | AAGAGCTTACCATGGGCCAG | TTGAAGGCTGCTCTAGGGTG | ChIP-qPCR |
| BCL3(123) | GGAAATCCCTTCCCGCAGAA | AGCGGAAGGGGTTAAGGTTG | ChIP-qPCR |
| BCL3(146) | AAATCCCTTCCCGCAGAACT | CCCGTTCCGGTAAAGTCTCA | ChIP-qPCR |
| MGP(234) | AGGCAAGAGAACTACAGGGC | TTCCAAAATTTATCTGCTGTGCT | ChIP-qPCR |
| MGP(299) | GGAGGGCAGCAGAAAACTTTG | AGCTTTTCCCTACCCCAGTTG | ChIP-qPCR |

| Gene | Forward primer (5'->3') | Reverse primer (5'->3') |
| --- | --- | --- |
| BCL3(RUNX1-negative) | AGGATTTTCCGAGCACCCAC | CCCTCTTCCATCTCTGCCAC |
| MGP(RUNX1-negative) | AGATGCATATCCCCAGGCAAA | TGGGTTTGGTCCACAGACAG |
| POSTN(RUNX1-negative) | CAGCCTTTACCCCCTTGTGA | TTTCCAGGAAGCATCGGCAA |
