# Supplementary material for: RUNX1 contributes to the mesenchymal subtype of glioblastoma in a TGFβ pathway-dependent manner
Source: Cell Death Dis. 2019 Nov 21;10(12):877. doi: 10.1038/s41419-019-2108-x (PMC6872557; doi:10.1038/s41419-019-2108-x)
Supplement: Supplementary file 2 — Supplementary materials and methods [file 41419_2019_2108_MOESM2_ESM.docx]

**Supplementary materials and methods.** The following antibodys were used in IHC assay: anti-RUNX1/AML1 (ab23980, Abcam), anti-RUNX1/AML1 (ab189172, Abcam), anti-BCL3 (ab27780, Abcam), anti-MGP (ab86233, Abcam), anti-periostin (ab14041, Abcam), anti-MXI1 (ab28740, Abcam) and anti-MMP9 (10375-2-AP, Proteintech). The following antibodys were used in Western blotting and co-IP assay: anti-FLAG (#14793, Cell Signaling Technology), anti-FLAG (#8146, Cell Signaling Technology), anti-p-SMAD3 (#9520, Cell Signaling Technology), anti-KMT1A / SUV39H1 (ab12405, Abcam), anti-CBFb (ab133600, Abcam), anti-collagen III (ab6310, Abcam), anti-GAPDH (60004-1-Ig, Proteintech), anti-GAPDH (10494-1-AP, Proteintech), anti-histone H3 (#4499, Cell Signaling Technology) and anti-tri-methyl-histone H3(Lys9) (#13969, Cell Signaling Technology). **Cell proliferation and cell adhesion Assays**  Cell proliferation was measured by the Cell Counting Kit-8 (CCK-8, Dojindo, Japan) assay. Briefly, cells were seeded at a density of 5 × 1000 cells per well in 96-well plates. Cell viability was assessed using CCK-8 at 24, 48, and 72 h after transfection. Absorbance was measured at 450 nm with a plate reader. For the cell adhesion assay, Matrigel was formulated into a 0.04 ug/ul artificial basement membrane gel using serum-free medium. Matrigel was placed in each well of a 96-well plate and allowed to solidify overnight. We added serum-free cell culture medium to each well allowed it to stand 60-90 min, and then washed off the excess glue, and seeded the cultured tumor cells at 40 000 cells/well. Three replicate wells were prepared per plate, and the plates were incubated in a 5% CO_2_ incubator at 37^o^C. The cells were incubated for 30, 60 and 120 min. The liquid was aspirated, and CCK-8 was then measured. **Immunohistochemistry, HE staining and Quantitative real-time PCR** Histological sections of tumor xenografts and patient tumor tissues were excised and fixed in 10% neutral buffered formalin. The assays were performed according to an established protocol, and the slides were analyzed using the National Institutes of Health (NIH) ImageJ software. Total RNA was extracted using TRIzol reagent (Invitrogen) according to the manufacturer’s instructions. cDNA was synthesized using a PrimeScript RT Reagent Kit (TaKaRa, Tokyo, Japan) according to the manufacturer's instructions. Real-time PCR amplification was performed by SYBR PremixExTaq (TaKaRa, Japan) on Hard-Shell PCR Plates (Bio-Rad). The relative quantity of each target gene was normalized using an endogenous control (GAPDH). qPCR and analyses were performed using a CFX Connect Real-Time PCR Detection System (Bio-Rad). PCR primers were designed and synthesized using a primer design tool (http://www.ncbi.nlm.nih.gov/tools/primer-blast/), and the primer sequences are listed in Table S7.
