# Supplementary figures and images for: RUNX1 contributes to the mesenchymal subtype of glioblastoma in a TGFβ pathway-dependent manner
Source: Cell Death Dis. 2019 Nov 21;10(12):877. doi: 10.1038/s41419-019-2108-x (PMC6872557; doi:10.1038/s41419-019-2108-x)

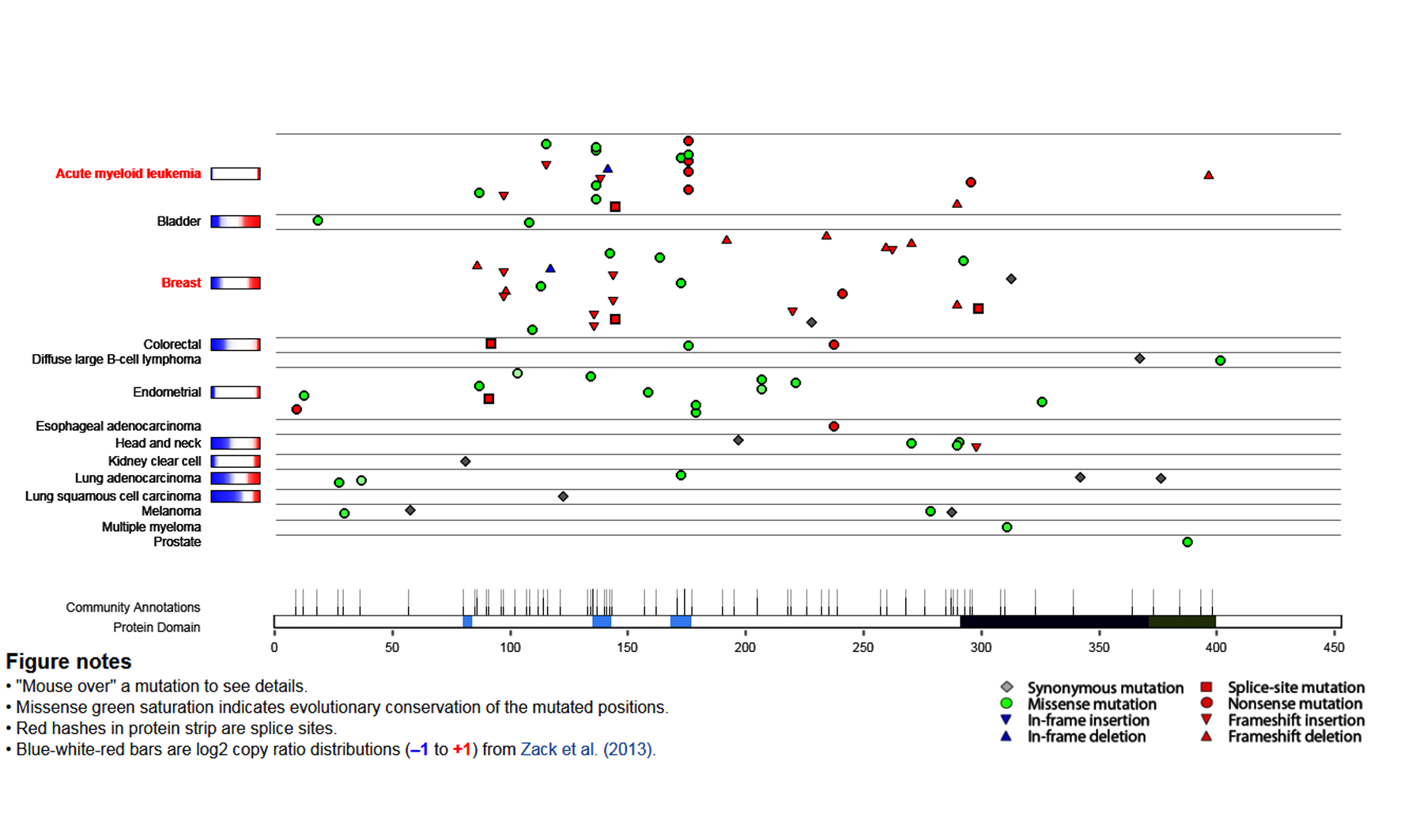

Supplement: Supplementary file 3 — figure s1 [file 41419_2019_2108_MOESM3_ESM.tif]

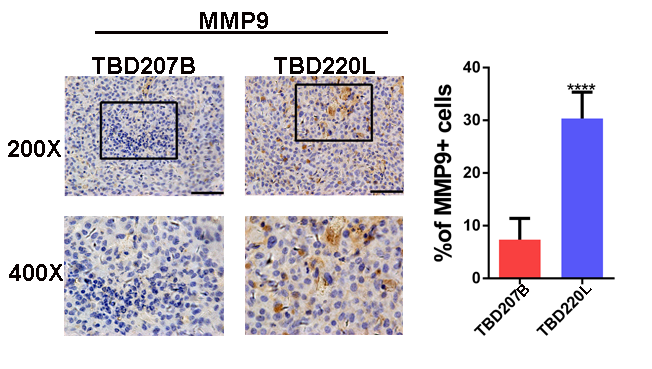

Supplement: Supplementary file 4 — figure s2 [file 41419_2019_2108_MOESM4_ESM.tif]

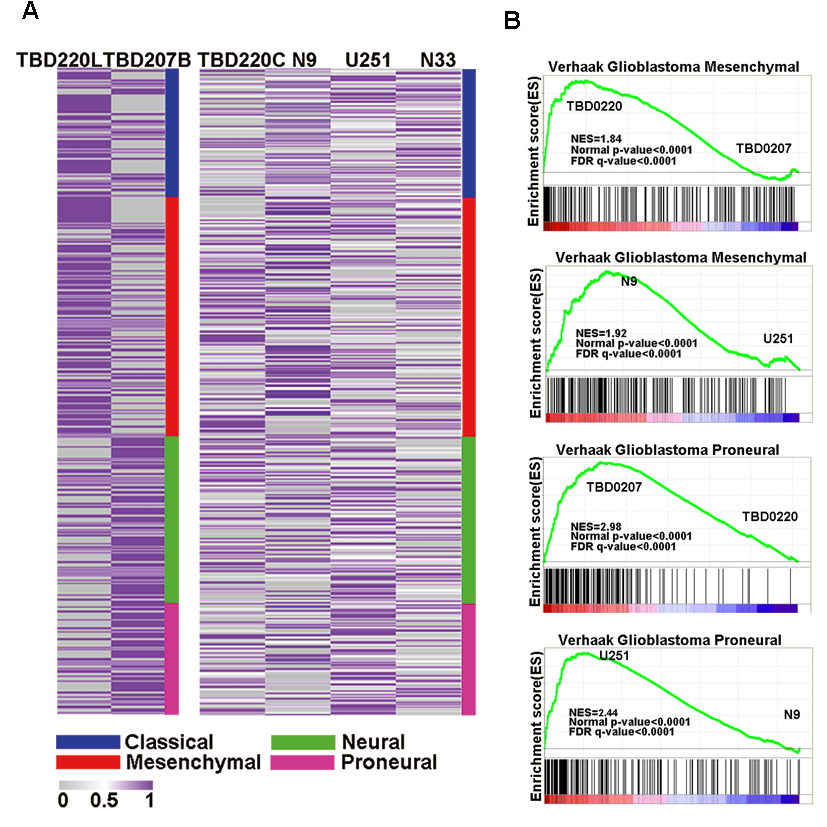

Supplement: Supplementary file 5 — figure s3 [file 41419_2019_2108_MOESM5_ESM.tif]

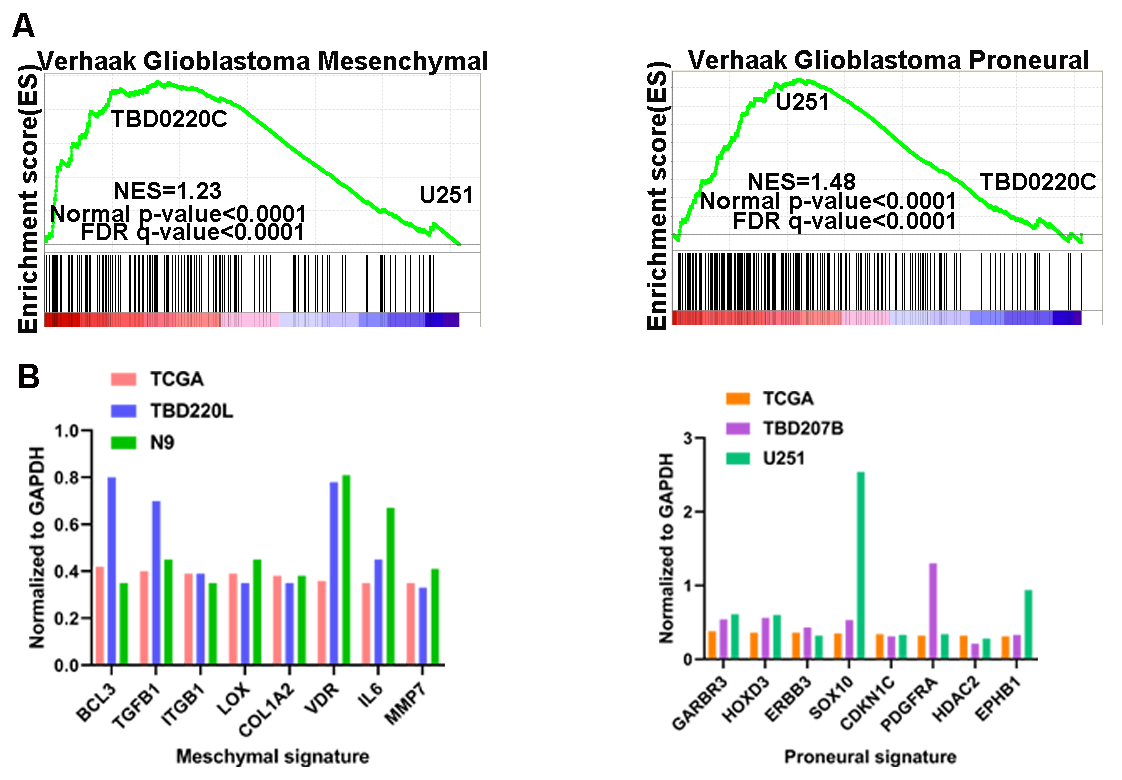

Supplement: Supplementary file 6 — figure s4 [file 41419_2019_2108_MOESM6_ESM.tif]

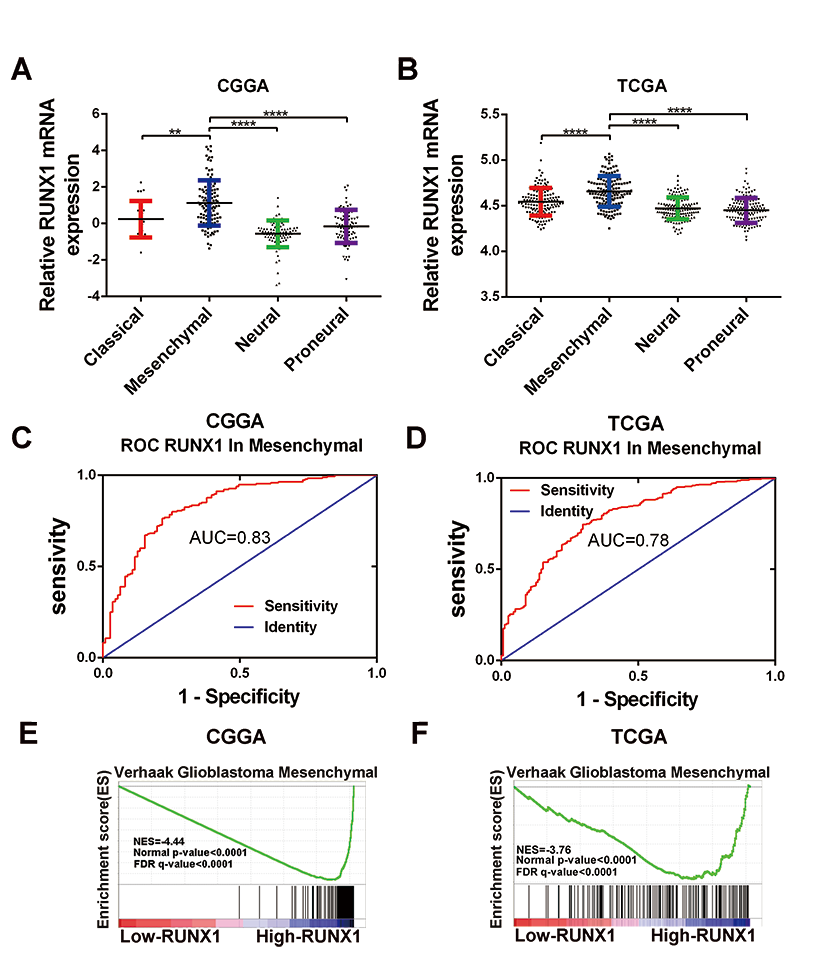

Supplement: Supplementary file 8 — figure s6 [file 41419_2019_2108_MOESM8_ESM.tif]

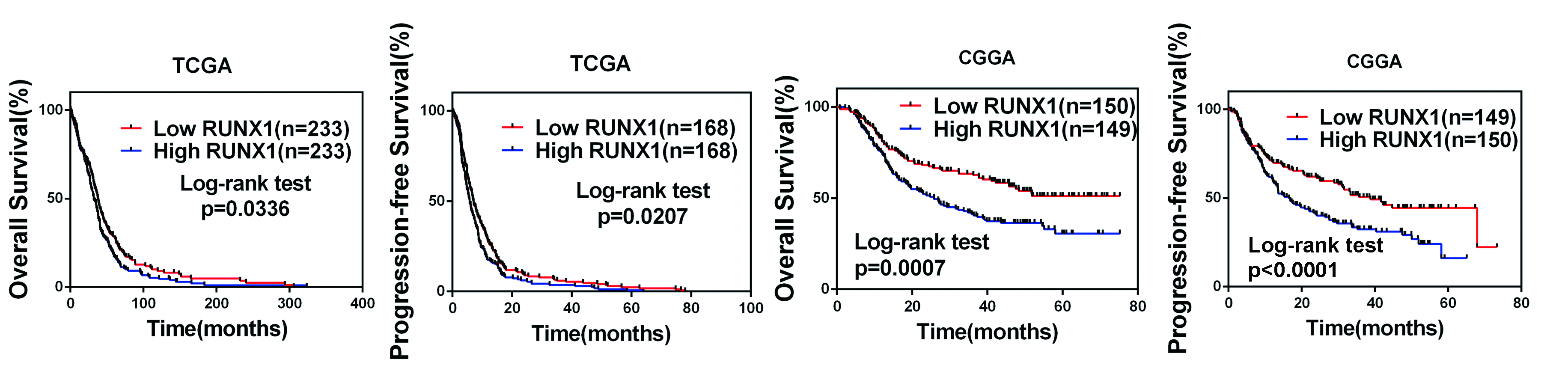

Supplement: Supplementary file 9 — figure s7 [file 41419_2019_2108_MOESM9_ESM.tif]

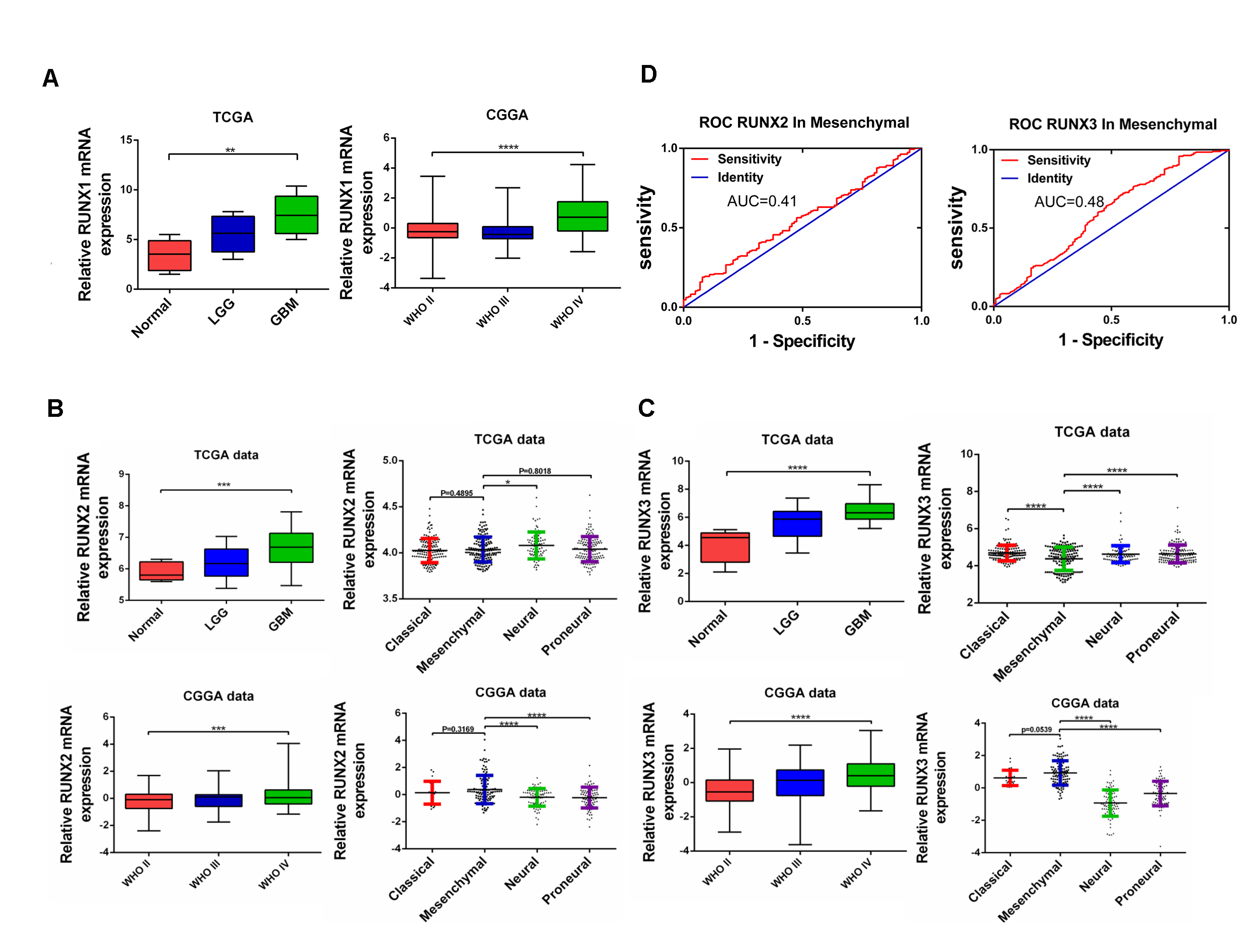

Supplement: Supplementary file 10 — figure s8 [file 41419_2019_2108_MOESM10_ESM.tif]

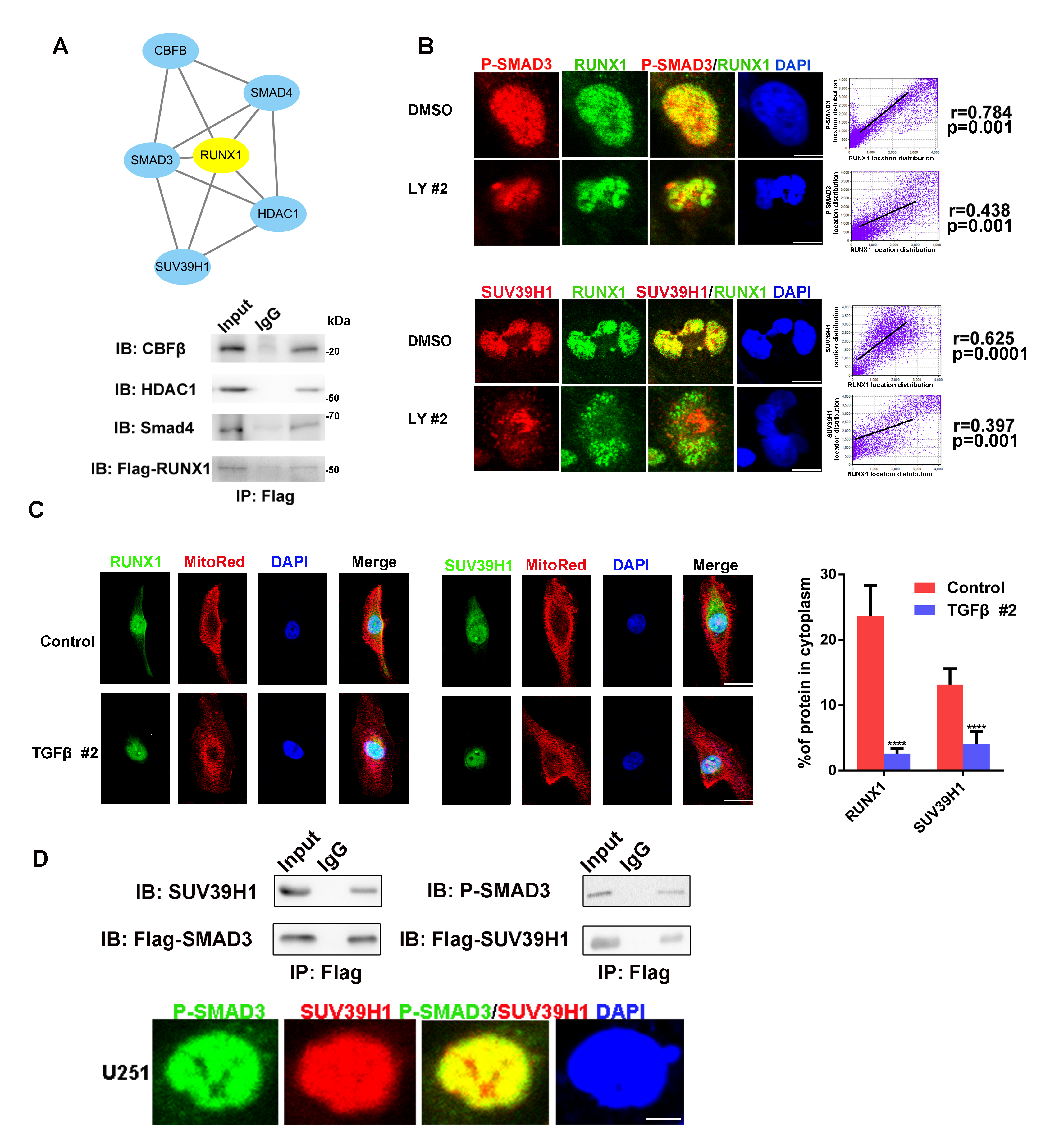

Supplement: Supplementary file 11 — figure s9 [file 41419_2019_2108_MOESM11_ESM.tif]

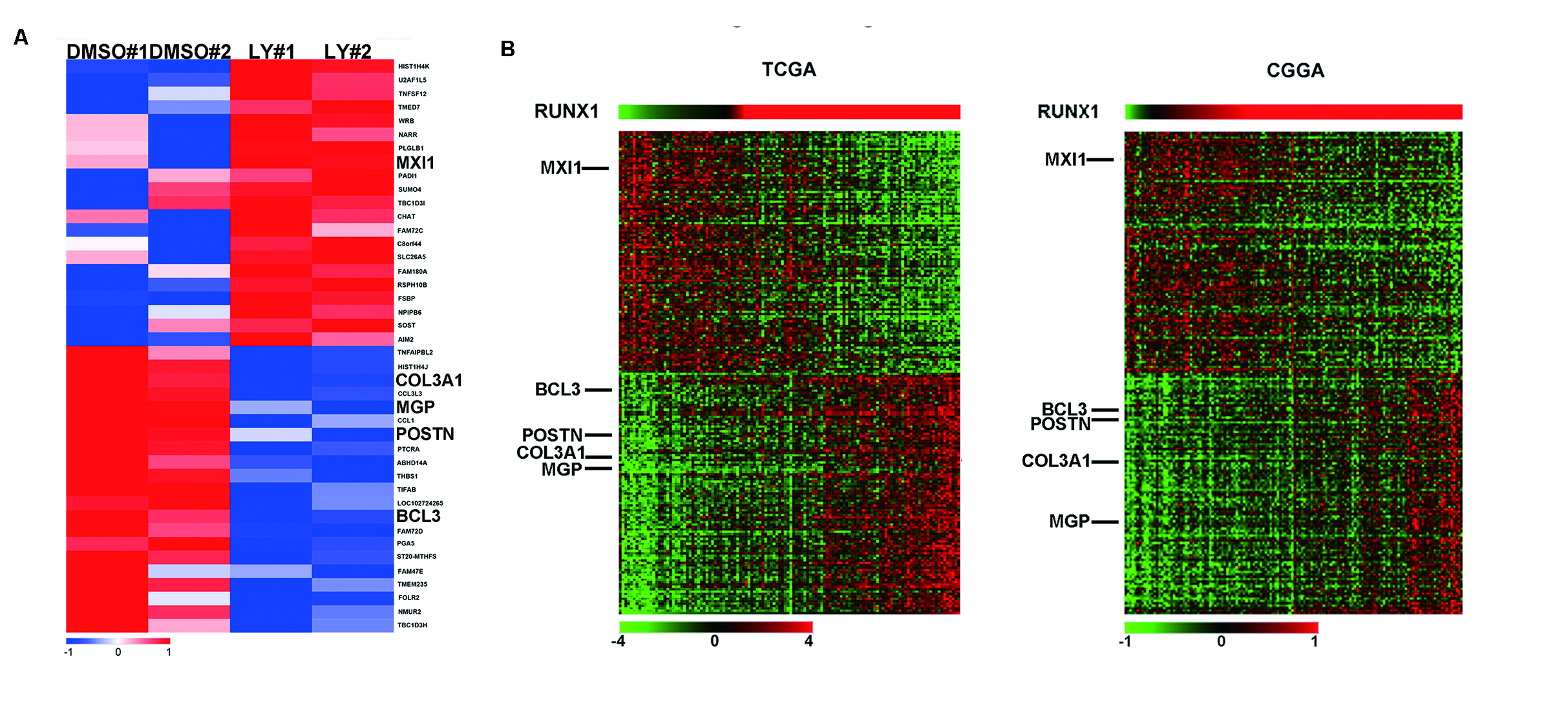

Supplement: Supplementary file 12 — figure s10 [file 41419_2019_2108_MOESM12_ESM.tif]

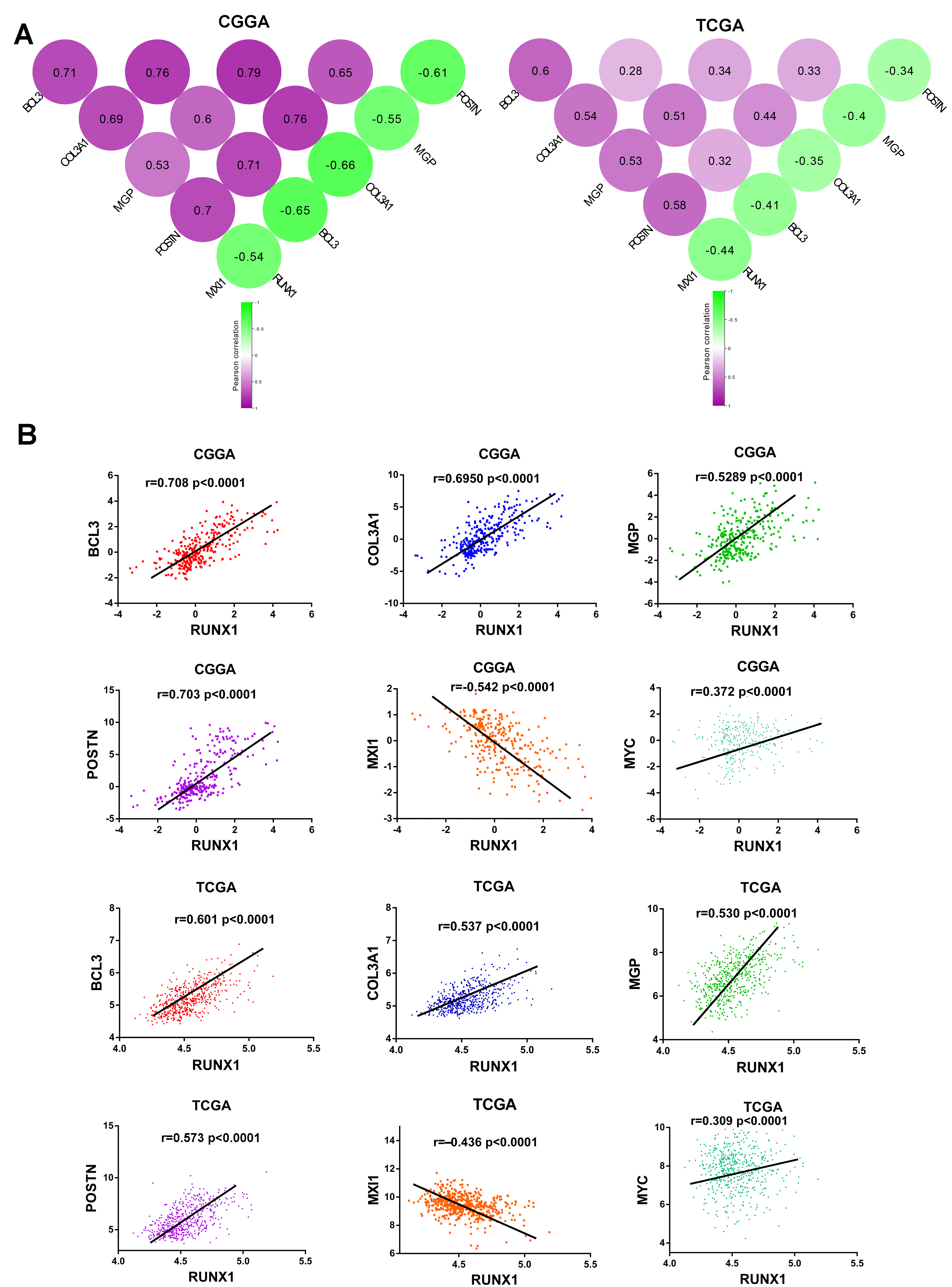

Supplement: Supplementary file 13 — figure s11 [file 41419_2019_2108_MOESM13_ESM.tif]

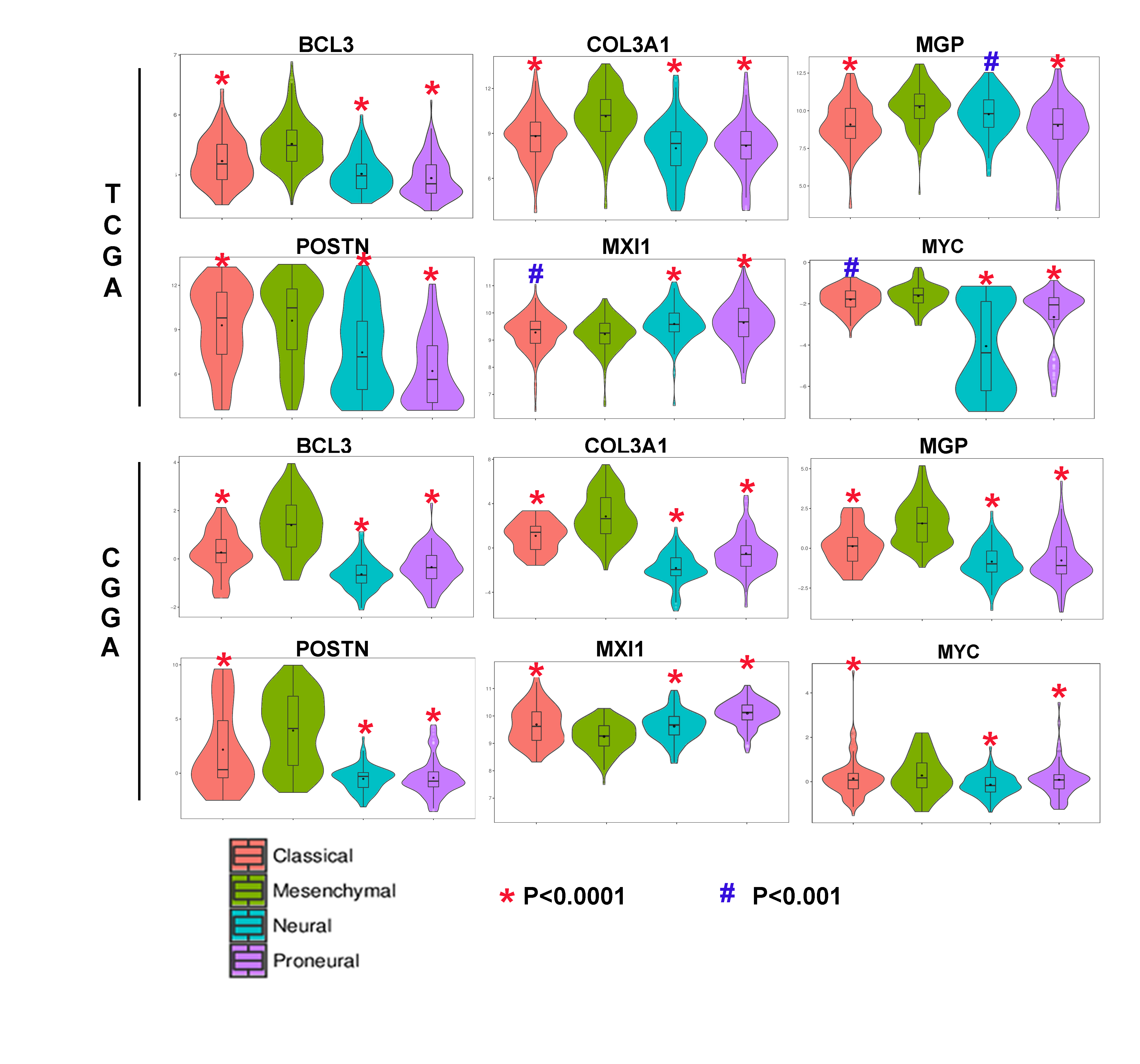

Supplement: Supplementary file 14 — figure s12 [file 41419_2019_2108_MOESM14_ESM.tif]

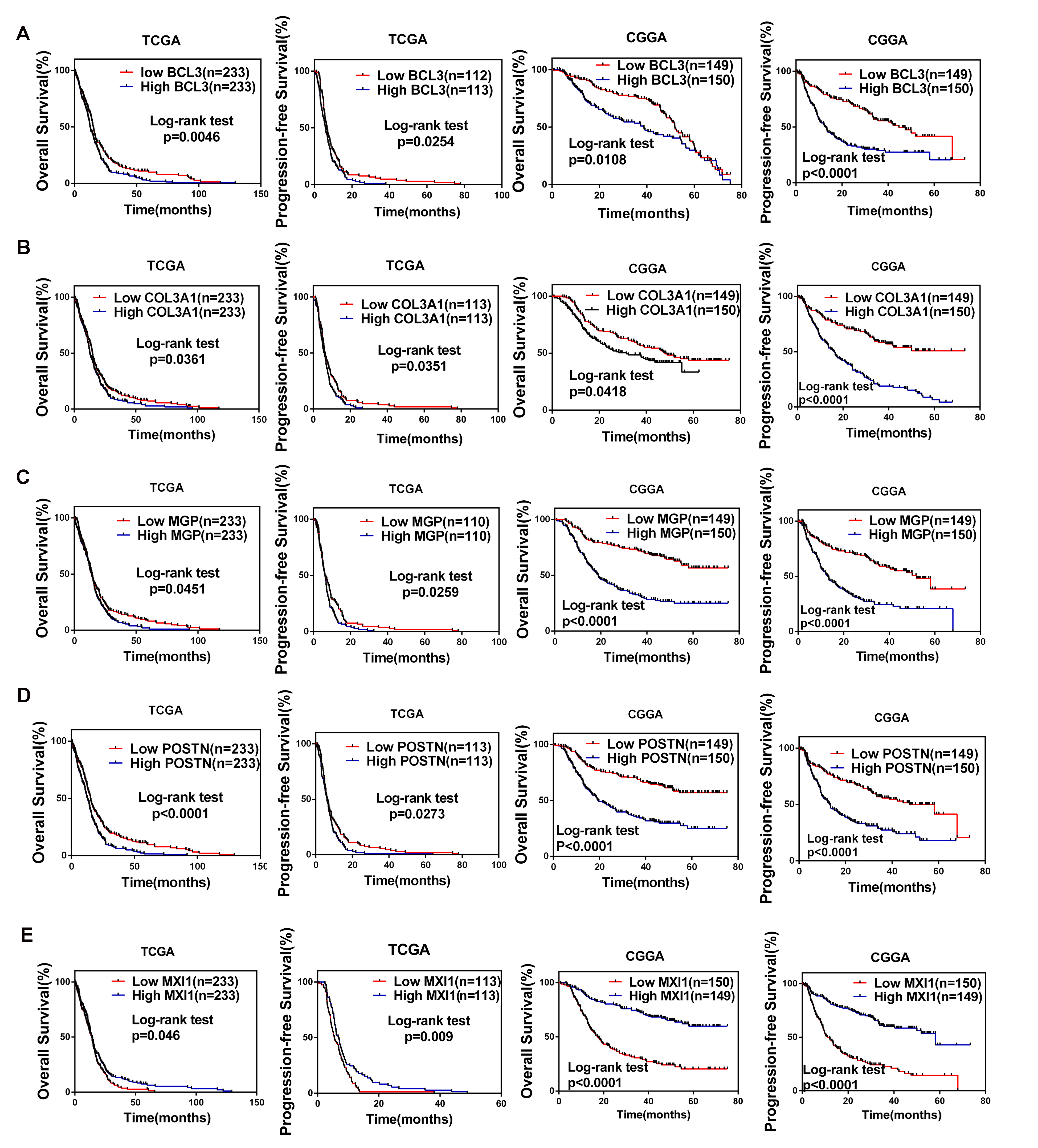

Supplement: Supplementary file 15 — figure s13 [file 41419_2019_2108_MOESM15_ESM.tif]

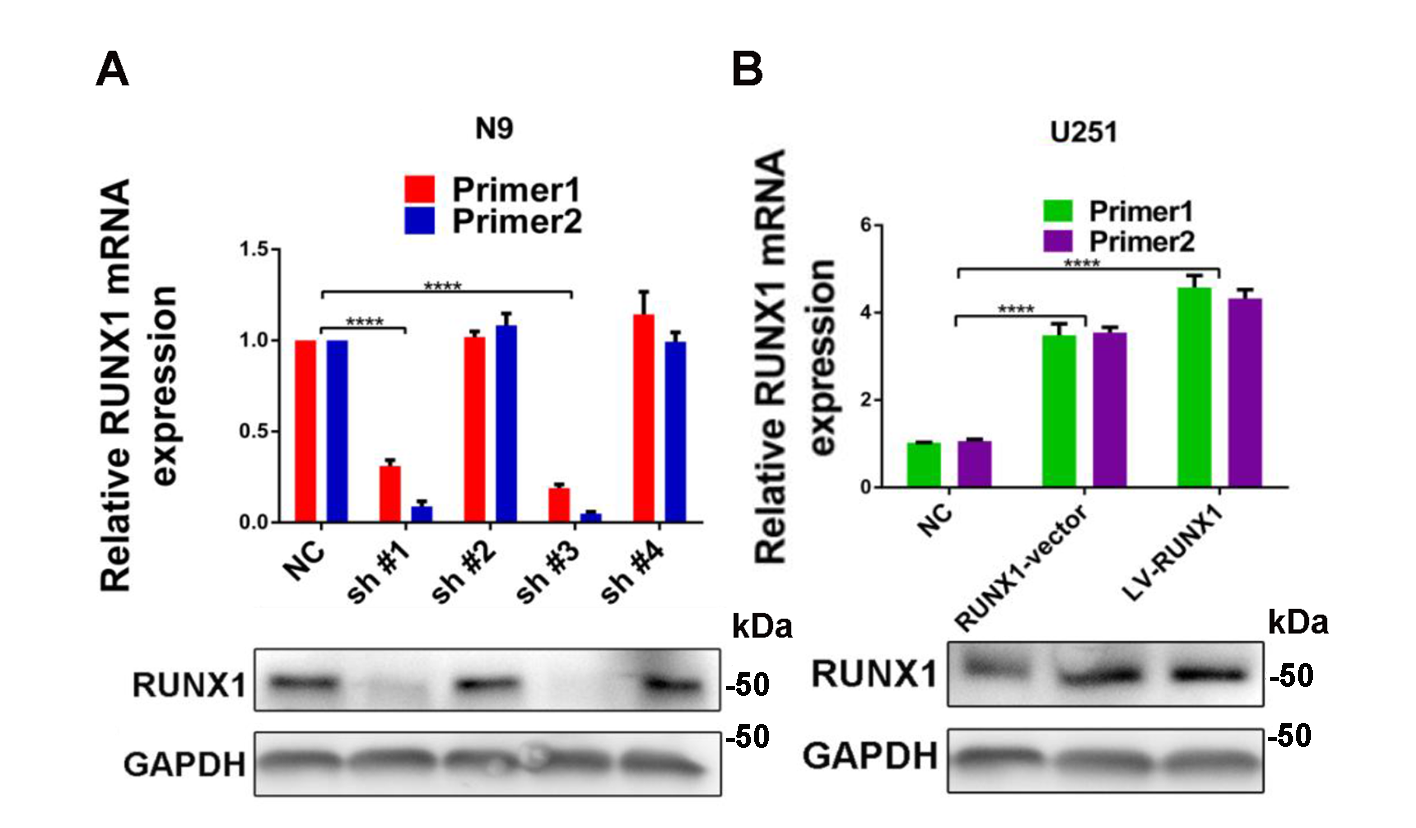

Supplement: Supplementary file 16 — figure s14 [file 41419_2019_2108_MOESM16_ESM.tif]

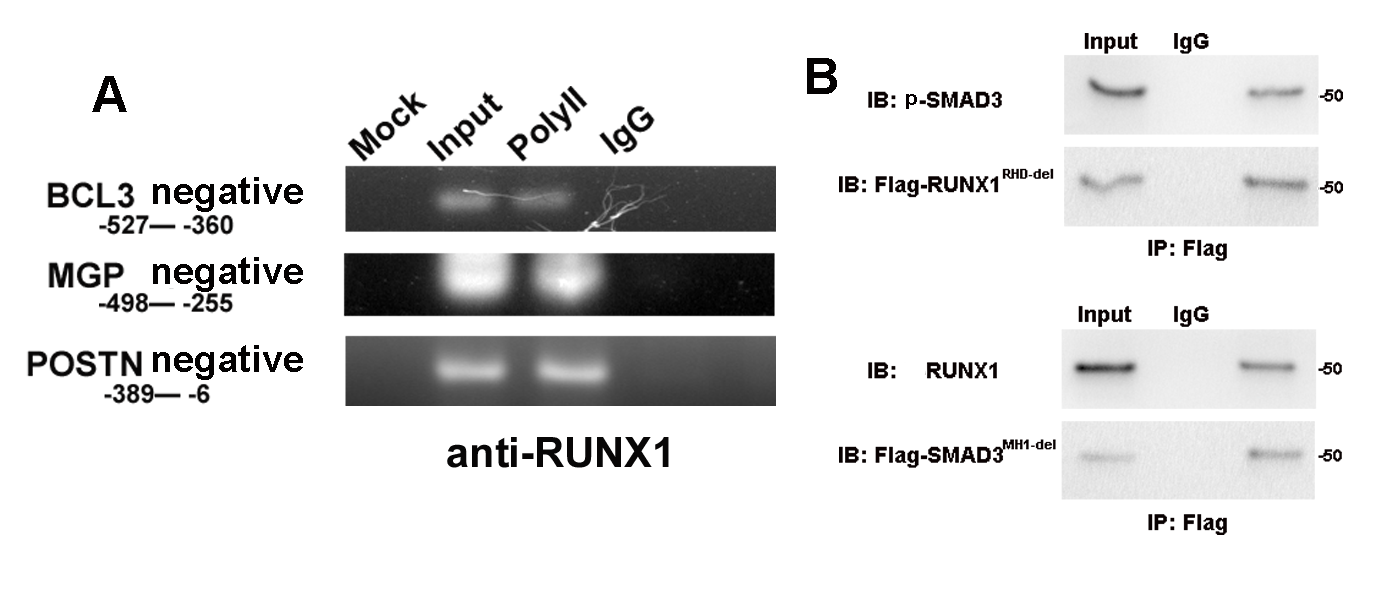

Supplement: Supplementary file 17 — figure s15 [file 41419_2019_2108_MOESM17_ESM.tif]

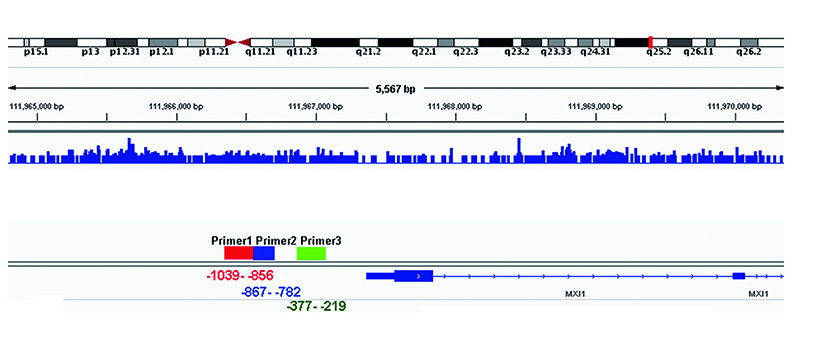

Supplement: Supplementary file 18 — figure s16 [file 41419_2019_2108_MOESM18_ESM.tif]

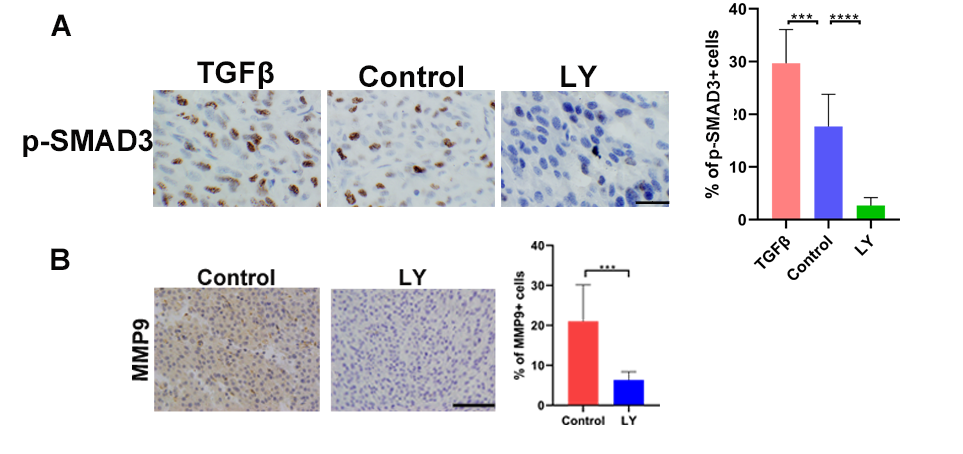

Supplement: Supplementary file 19 — figure s17 [file 41419_2019_2108_MOESM19_ESM.tif]
