# Supplementary material for: RUNX1 contributes to the mesenchymal subtype of glioblastoma in a TGFβ pathway-dependent manner
Source: Cell Death Dis. 2019 Nov 21;10(12):877. doi: 10.1038/s41419-019-2108-x (PMC6872557; doi:10.1038/s41419-019-2108-x)
Supplement: Supplementary file 20 — Supplementary figure legends [file 41419_2019_2108_MOESM20_ESM.docx]

**Fig. S1.** RUNX1 mutations in the TCGA pan-cancer database.

**Fig. S2.** IHC were performed to analysis the expression of MMP9 in TBD0207B and TBD0220L tissue sites. Quantitative analyses were performed using ImageJ software for each high-magnification view. n=3 per group. Scale, 100 μm. (**** indicate p<0.0001).

**Fig. S3. a.** mRNA expression levels of molecular markers of the classical, Mes, neural and proneural subtypes in TBD0220L, TBD0207B, TBD0220C, N9, U251 and N33 cells. **b.** A comparison of mRNA expression levels in VerhaakMes phenotyping packages (TBD0220L vs TBD0207B and N9 vs U251) and VerhaakProneural phenotyping packages (TBD0207B vs TBD0220L and U251 vs N9).

**Fig. S4.** **a.** GSEA for comparison of mRNA expression levels in Verhaak Mes phenotyping packages (TBD0220C vs U251) and Verhaak Proneural phenotyping packages (U251 vs TBD0220C). **b.** The expression of marker gene in TBD0207B, U251, TBD0220L or N9 and the sample that has been typed by TCGA.

**Fig. S5.** Mutual correlations in mRNA expression in the N9, TBD0220, TBD0220C, U251, N33 and TBD0207 tissues or cell lines.

**Fig. S6.** The relative expression of RUNX1 was highest in the Mes group in the CGGA **(a)** and TCGA **(b)** datasets. ROC curves were used to evaluate the sensitivity of RUNX1 as a molecular marker of Mes GBM in the CGGA **(c)** and TCGA **(d)** datasets. Genes that were positively correlated with RUNX1 presented Verhaak Mes signatures in the CGGA **(e)** and TCGA **(f)** datasets. (** and **** indicate p<0.01 and p<0.0001, respectively).

**Fig. S7.** The overall survival and progression-free survival about RUNX1 in the TCGA and CGGA datasets were shown.

**Fig. S8.** **a.** The expression levels of RUNX1, RUNX2 and RUNX3 are shown for different WHO grades **(b)** and GBM subtypes **(c)**. **d.** ROC curves were used to evaluate the sensitivity of RUNX2 and RUNX3 as the molecular markers of Mes GBM in CGGA datasets. (*, **, *** and **** indicate p<0.05, p<0.01, p<0.001 and p<0.0001, respectively).

**Fig. S9. a.** The string protein-protein interaction network of RUNX1. The interaction between predictive protein and RUNX1 were validated by co-IP. **b.** Subcellular localization of RUNX1, p-Smad3 and SUV39H1 in N9 cells under normal and LY2109761-treated conditions. Scale bar, 5 μm. A correlation analysis verified positional overlap. n=5 per group. **c.** Localization of RUNX1 and SUV39H1 in U251 cells under normal or TGFβ-treated conditions. ImageJ calculated the proportion of RUNX1 and SUV39H1 in the cytoplasm before and after TGFβ treatment. Scale bar, 10 μm. **d.** The interaction between SUV39H1 and P-Smad3 was validated by co-IP. Subcellular localization of p-Smad3 and SUV39H1 in 293T cells. Scale bar, 5 μm. (**** indicate p<0.0001).

**Fig. S10.** **a.** Hierarchical cluster analysis and heatmap of the top 42 most significantly enriched genes in a comparison of sequencing results between DMSO and LY2109761 treated PDX. **b.** Hierarchical cluster analysis and heatmap of the top 200 most significantly RUNX1-associated enriched genes.

**Fig. S11. a.** Mutual correlation of RUNX1, BCL3, COL3A1, MGP, POSTN and MXI1 in the Mes samples of TCGA and CGGA datasets. The number in the circle represents the r value. **b.** Correlation analysis of RUNX1 expression with the expression of BCL3, COL3A1, MGP, POSTN, MXI1 and MYC in the Mes samples of TCGA and CGGA datasets. (The TCGA microarray data was log-transformed.)

**Fig. S12.** The mRNA expression levels of BCL3, COL3A1, MGP, POSTN, MXI1 and MYC in GBM molecular subtypes.

**Fig. S13. a-e.** The overall survival and progression-free survival about BCL3, COL3A1, MGP, POSTN and MXI1 in the TCGA and CGGA datasets were shown.

**Fig. S14. a-b.** Western blotting and q-PCR were used to analyze the knockdown or overexpression efficiency of the lentivirus and plasmids. GAPDH was used as the loading control. (**** indicate p<0.0001).

**Fig. S15. a.** ChIP analysis of the ability of RUNX1-negative region to bind to the BCL3, MGP or POSTN promoter using antibodies against RUNX1 in 293T cells. **b.** The interaction between p-Smad3 and Flag-RUNX1RHD-del or RUNX1 and Flag-Smad3MH1-del were validated by co-IP.

**Fig. S16.** H3K9me3 was enriched at the promoter region of MXI1 in the GSE103408 dataset. “1”, “2”, and “3” indicate the positions of the primer extension sequences.

**Fig. S17. a.** IHC was used to analyze the expression of P-Smad3 at the control, TGFβ and LY2109761 sites. Quantitative analyses were performed using ImageJ software for each high-magnification view. n=5 per group. Scale, 20 μm. **b.** IHC was used to analyze the expression of MMP9 at the control and LY2109761 sites. Quantitative analyses were performed using ImageJ software for each high-magnification view. n=5 per group. Scale, 100 μm. (*** and **** indicate p<0.001 and p<0.0001).
