# Supplementary material for: RUNX1 contributes to the mesenchymal subtype of glioblastoma in a TGFβ pathway-dependent manner
Source: Cell Death Dis. 2019 Nov 21;10(12):877. doi: 10.1038/s41419-019-2108-x (PMC6872557; doi:10.1038/s41419-019-2108-x)
Supplement: Supplementary file 21 — table s2 [file 41419_2019_2108_MOESM21_ESM.docx]

Table S2. GO and KEGG pathway analysis

| Category | Term | Count | P Value |
| --- | --- | --- | --- |
| 539 samples |  |  |  |
| GOTERM_BP_DIRECT | GO:0030198~extracellular matrix organization | 10 | 8.52E-08 |
| GOTERM_BP_DIRECT | GO:0007155~cell adhesion | 10 | 5.32E-06 |
| GOTERM_CC_DIRECT | GO:0031012~extracellular matrix | 12 | 2.71E-08 |
| GOTERM_MF_DIRECT | GO:0002020~protease binding | 5 | 2.15E-04 |
| KEGG_PATHWAY | hsa04512:ECM-receptor interaction | 8 | 7.02E-06 |
| 483 samples |  |  |  |
| GOTERM_BP_DIRECT | GO:0007155~cell adhesion | 15 | 8.86E-06 |
| GOTERM_BP_DIRECT | GO:0051052~regulation of DNA metabolic process | 7 | 0.0013 |
| GOTERM_CC_DIRECT | GO:0015629~actin cytoskeleton | 5 | 7.92E-04 |
| GOTERM_MF_DIRECT | GO:0008092~cytoskeletal protein binding | 13 | 1.51E-05 |
| KEGG_PATHWAY | hsa04510:Focal adhesion | 7 | 8.23E-04 |
| Rembrandt |  |  |  |
| GOTERM_BP_DIRECT | GO:0030198~extracellular matrix organization | 11 | 8.43E-04 |
| GOTERM_CC_DIRECT | GO:0007155~cell adhesion | 11 | 0.0052 |
| GOTERM_MF_DIRECT | GO:00004872~receptor activity | 6 | 0.0013 |
| KEGG_PATHWAY | hsa04512:ECM-receptor interaction | 7 | 0.0017 |
| CGGA |  |  |  |
| GOTERM_BP_DIRECT | GO:0030198~extracellular matrix organization | 5 | 3.02E-08 |
| GOTERM_BP_DIRECT | GO:0007155~cell adhesion | 6 | 9.71E-04 |
| GOTERM_CC_DIRECT | GO:0043062~extracellular matrix structure constituent | 4 | 7.97E-09 |
| GOTERM_MF_DIRECT | GO:0009611~response to mechanical stimulus | 4 | 1.56E-10 |
| KEGG_PATHWAY | hsa00230:Purine metabolism | 3 | 0.017818 |
