# Supplementary material for: RUNX1 contributes to the mesenchymal subtype of glioblastoma in a TGFβ pathway-dependent manner
Source: Cell Death Dis. 2019 Nov 21;10(12):877. doi: 10.1038/s41419-019-2108-x (PMC6872557; doi:10.1038/s41419-019-2108-x)
Supplement: Supplementary file 22 — table s3 [file 41419_2019_2108_MOESM22_ESM.docx]

Table S3. Luciferase plasmids

|  | Insertion sequence |
| --- | --- |
| BCL3-WT | GGTACCCCTAAGCCTCGCCCCTCCAAACCTCAGCTTTCCTTTCTGTGCATTGGGATAGTGCTCCACACCCCTCTCCTGTGTCTGAGGGTCCCCAGGGGTGGGGTTGGATGGGCAGGGAACACAGCCCCCAGCCTGTCCAACCGAATTCCTCCCCTCTTCCCTTGCTGTGGCCCCCACAACACCAGCAGGCAGGGGTTGCCATTGGTCCCATTTTCCAGAAGAACAAACTGAGTCTCAAGGCAGGGGATGGCTTGTCTGAGGTCCCACCGGGAGCAAGGGGTGGAGGTGGAATTAACACAGGTCTGTGCTCGGGATGTTTCGCCATGAAGGGGGATAAGGGAGATCCAGAGAGAGGTCGAAAGGAAGGGAAGGGGTGAGGCCTCTCCACACAGCCACTTCTCACATCCTGGGTTAACGATCCACTCGGGTGGGGCCCGAGTCCGGCCAAAGTCCCTTCCCGGGGCCCCAATACCCACTTTCAAGGTCCCAGGGGACACAGAGACAGCAACAGAGATGGGGAGACAGAGACAGAGGGCGGGGCAGGAGACAACCAGAGGGAGGTCAGGCAAGTTTCGAAGACAGAAGGAGACCGGGGGAGAGAACCAGAGAGACGCCGCGTGCCCCCTGGGCAAGTCCGGGGTGCAGCCCCCATGCCCCGCCCCCTGTGTCCCCAAAGCCTCCCTCTCCGCCTCCTGTGACTCAGTGACCCGGACTCAACCCCAGCGGCTGCCCCGCCCCCAGGGAAATCCGGGAAAGCCCCGGACCTTCGGGGCCCCACCCCGGGGCGGGGAGAGGGAGAGGAGGGGAGGAGACGGGGAAGAGTCCCGCCTCCCTCCTCCCTCCCTCCCATCTCCAGCCTGAGTCATGCCCCCAACCCGCCTAGCCTGTTCCCCACCCATCAACCCCTCCTCGAGGTGGGAGGGGAAGTCGGGGGGAAGCCAAACTGCTCCCCGCTCCTGCAGCACCGGCCTCGGTCGCGCTGACTCTGGCCTGGTGTCCGTGTCTCTTGCTATCTCTCTTTCTCTCAAGATCTCTGCGCCTGTCTCCATGTCTCTTTCTCTCTGTGCACCTAGGATTTTCCGAGCACCCACCCCGGTGCCCCGCGGGCCCCGGCTGGGGGCAGGGCCCCCAACGAGTGCAGAGACACAATCAGTGGAGCGCTCCCCACCCTCACCCCACCCCCAGCCCCTTTAGACCCACAGCTGATGAGGCACGTGGAGTGGCAGAGATGGAAGAGGGGAGGGAGAGGAATCGTTCCTGGCCGCCTGCCGAGTGCCAGTCCCTGCCCCGGGTGCGTCTCTTCACTCCCACTAAGGAGGAAACGGCTCAGAGAGGGGAAGTGTTTGGCCAAGGTCACCCAGCGAGTAAGTGGGAACCGAGATTCCAAACCCCGTCCCAGAGATGCCAAGGCCTCCTGAGAGACAGAGAAGCAGACAGCAACTGAGAGGCAGAGAGATGGTGACAGACACAAAGAGACAAAAGAGAGAGACAGAGACGGAACAGAGCACACAGAGACAAACGCGGGGTTGCGGAGAGAAACACCTACTCAGACAGGAGAACCAGAGAGACAGTTACAGACTCAGAGATAGAGATGTTGAGAGATAGGGCCAGAAAGACAAAAACAGAGGCAGAGAGAGCGGCCCTTGGCAGCAGGGGTGGGGACACCCCCCCACCCCCCGACCCCGCCTCCTCTCCCCCCACCCCTCCTTTCCTCTCCCTCCCCCGCCGAGGCCTGGCTGCCCCAGGCGCCGCGGGCCGGGAGGGGGCAAGCGGGGCGCGGCGCGGGCGGGGCGCAGGGCAGGCTGCAAAGCTT |
| BCL3-MU | GGTACCCCTAAGCCTCGCCCCTCCAAACCTCAGCTTTCCTTTCTGTGCATTGGGATAGTGCTCCACACCCCTCTCCTGTGTCTGAGGGTCCCCAGGGGTGGGGTTGGATGGGCAGGGAACACAGCCCCCAGCCTGTCCAACCGAATTCCTCCCCTCTTCCCTGAGGAGAATTTCCACAACACCAGCAGGCAGGGGTTGCCATTGGTCCCATTTTCCAGAAGAACAAACTGAGTCTCAAGGCAGGGGATGGCTTGTCTGAGGTCCCACCGGGAGCAAGGGGTGGAGGTGGAATTAACACAGGTCTGTGCTCGGGATGTTTCGCCATGAAGGGGGATAAGGGAGATCCAGAGAGAGGTCGAAAGGAAGGGAAGGGGTGAGGCCTCTCCACACAGCCACTTCTCACATCCTGGGTTAACGATCCACTCGGGTGGGGCCCGAGTCCGGCCAAAGTCCCTTCCCGGGGCCCCAATACCCACTTTCAAGGTCCCAGGGGACACAGAGACAGCAACAGAGATGGGGAGACAGAGACAGAGGGCGGGGCAGGAGACAACCAGAGGGAGGTCAGGCAAGTTTCGAAGACAGAAGGAGACCGGGGGAGAGAACCAGAGAGACGCCGCGTGCCCCCTGGGCAAGTCCGGGGTGCAGCCCCCATGCCCCGCCCCCTGTGTCCCCAAAGCCTCCCTCTCCGCCTCCTGTGACTCAGTGACCCGGACTCAACCCCAGCGGCTGCCCCGCCCCCAGGGAAATCCGGGAAAGCCCCGGACCTTCGGGGCCCCACCCCGGGGCGGGGAGAGGGAGAGGAGGGGAGGAGACGGGGAAGAGTCCCGCCTCCCTCCTCCCTCCCTCCCATCTCCAGCCTGAGTCATGCCCCCAACCCGCCTAGCCTGTTCCCCACCCATCAACCCCTCCTCGAGGTGGGAGGGGAAGTCGGGGGGAAGCCAAACTGCTCCCCGCTCCTGCAGCACCGGCCTCGGTCGCGCTGACTCTGGCCTGGTGTCCGTGTCTCTTGCTATCTCTCTTTCTCTCAAGATCTCTGCGCCTGTCTCCATGTCTCTTTCTCTCTGTGCACCTAGGATTTTCCGAGCACCCACCCCGGTGCCCCGCGGGCCCCGGCTGGGGGCAGGGCCCCCAACGAGTGCAGAGACACAATCAGTGGAGCGCTCCCCACCCTCACCCCACCCCCAGCCCCTTTAGACCCACAGCTGATGAGGCACGTGGAGTGGCAGAGATGGAAGAGGGGAGGGAGAGGAATCGTTCCTGGCCGCCTGCCGAGTGCCAGTCCCTGCCCCGGGTGCGTCTCTTCACTCCCACTAAGGAGGAAACGGCTCAGAGAGGGGAAGTGTTTGGCCAAGGTCACCCAGCGAGTAAGTGGGAACCGAGATTCCAAACCCCGTCCCAGAGATGCCAAGGCCTCCTGAGAGACAGAGAAGCAGACAGCAACTGAGAGGCAGAGAGATGGTGACAGACACAAAGAGACAAAAGAGAGAGACAGAGACGGAACAGAGCACACAGAGACAAACGCGGGGTTGCGGAGAGAAACACCTACTCAGACAGGAGAACCAGAGAGACAGTTACAGACTCAGAGATAGAGATGTTGAGAGATAGGGCCAGAAAGACAAAAACAGAGGCAGAGAGAGCGGCCCTTGGCAGCAGGGGTGGGGACACCCCCCCACCCCCCGACCCCGCCTCCTCTCCCCCCACCCCTCCTTTCCTCTCCCTCCCCCGCCGAGGCCTGGCTGCCCCAGGCGCCGCGGGCCGGGAGGGGGCAAGCGGGGCGCGGCGCGGGCGGGGCGCAGGGCAGGCTGCAAAGCTT |
| MGP-WT | GGTACCGTATAAAACTTTTATCTATGGCTAGCTTGTCCCCCCAAAGTCATGCAATATAGTGAACTGGCTTTCGCACTTTAAATTATTCATTGATCATGTAATGATTCAGATGATTCATCTTCCAAGATGGACACTGAAACTAACACTCATAGTAGGTTGTGGTTTAAAGAGTGGAACAACCGCCAGTCTCATTAGTGGAAATTGTGATGGTTGAATTTATCAAGGATGAACATACACGGTCTTCTTTCTGAGATTTTCTTTAAGATTTTCGCACAGATAATCTATTTCTTAGGTTTTGGAGAGAAAACTTGAATTTTATTGATCCCTCAGAACTCAATCTTTCAGATTTCAAAGGAGCTATTTCTTTTAATGGGGACTCTGTTAATATTTATAAAAGCTCTTCACAGGATGGAGGGTGGGAGGGAAACTCCATCCCAACAAGACAAAAAGAATGAAGCATGAGGCTCCACCTAGTTCATCACTGCTCCTTGAAATACATCAGTATTGAAAGACACATCCACCCCACCCCCAACCCAGCCCTATTGCTGTTCCAGCTCAAGAGTCAGAGGTCCCGAAGCTGTAGCTCTTCTACAATCTGCTGCTCTGTGACTTCAAGTCTGTTGTCTGCAAAGAAAACTATTGGGTTCCCAAGCAAGAGAGGCACATCTGGTAGGACAGATTTTGTGATTGCAAAAGAAGGGGGAAAAAAAGAAAGAAAGAAAAGACCTCTCTATACAAGATAACCAGAGGCATCAAACTGAAATCCTCCTGTGGAAAATAAGCTAGTACTTCTGGGCCTGATGGTGTAGTGAAAACCTGTGCTTGAGGATACATTACAGTGAAAGAGCAAAGTGAATAGTAAGTAGCTATTACTTACCTCCTTAGGGAGGTGTGTTGTTTGTCTGTACATCCCCCACAGCACCTAGCACAGTACCTTGCATCTCACCTGCCACTCACTAAAAAGTCTATCAAGTTAGTTAATTATCGAGACAACGCCCTCAGAAATGAGAGAACAGTACCCTCTTATCCTTGCTGCACTTTCCAGCACTGATACGCTGCCTAAAAGAGGACTAGGGCACAGGTTTGAATTAATGTCACAAAACTGGATGGGCAAGTTACAACGGTGTTGATTAAGGAAACAGAACTCATGGTGCACCGGATATCTCCATCCTGATGAACCCTTGGAAAAATGCCAAAGATGCATATCCCCAGGCAAATGCCTGATTAGTCTGGGATTGATAGATTGGTCTAGGATTCAGCCCTACTGGGAAGATGTCTAAATTATAATCAGTGTAGAAAGCGAAGTTCTCCTAGAAGAAGAGGCAAAGGTTAAAAAGAAGAAAAGAAAAGAAAGTGAAGTCCTTTCTCCCCCAAAACCTCTCATCAATCAATCAGGGTAACAAACAGAACACTAGGGCTCTGTCTGTGGACCAAACCCAAAAGCCCTGCGGTCAGGGCCAGGAGGGTAGATCATGTGTTTGTGGCAACTTCCTCTGTGGGCTTTTGCCCAGGTCTGTCCCCAAGCATACGATGGCCAAAACTTCTGCACCAGAGCAGCATCCTGTGTAACACAGTCAGGTCCAGCAGTTAGGGAAAACTGCCCACTCAGAGTAGATAATATCTGGAAGGAATGACTGTTTGGGAAAAGTTCCAATGCTAGTTCAGTGCCAACCCTTCCCCACCTTCTCCAGCTCTCTCCCACTGGTTCCTCCCCTCTCAACTGCTCTGGTTCTTATAAAAACCTCACAGCCTTCCACTAACATCCCGTAGGAGCCTCTCTCCCTACTGCTGCTACACAAGACCCTGAGACTGACCTGCAGGACGAAACCATGAAGAGCCTGATCCTTCTTGCCATCCTGGCCGCCTTAAGCTT |
| MGP-MU | GGTACCGTATAAAACTTTTATCTATGGCTAGCTTGTCCCCCCAAAGTCATGCAATATAGTGAACTGGCTTTCGCACTTTAAATTATTCATTGATCATGTAATGATTCAGATGATTCATCTTCCAAGATGGACACTGAAACTAACACTCATAGTAAAGGAGAAGGGAAAGAGTGGAACAACCGCCAGTCTCATTAGTGGAAATTGTGATGGTTGAATTTATCAAGGATGAACATACACGGTCTTCTTTCTGAGATTTTCTTTAAGATTTTCGCACAGATAATCTATTTCTTAGGTTTTGGAGAGAAAACTTGAATTTTATTGATCCCTCAGAACTCAATCTTTCAGATTTCAAAGGAGCTATTTCTTTTAATGGGGACTCTGTTAATATTTATAAAAGCTCTTCACAGGATGGAGGGTGGGAGGGAAACTCCATCCCAACAAGACAAAAAGAATGAAGCATGAGGCTCCACCTAGTTCATCACTGCTCCTTGAAATACATCAGTATTGAAAGACACATCCACCCCACCCCCAACCCAGCCCTATTGCTGTTCCAGCTTCAAGAGTCAGAGGTCCCGAAGCTGTAGCTCTTCTACAATCTGCTGCTCTGTGACTTCAAGTCTGTTGTCTGCAAAGAAAACTATTGGGTTCCCAAGCAAGAGAGGCACATCTGGTAGGACAGATTTTGTGATTGCAAAAGAAGGGGGAAAAAAAGAAAGAAAGAAAAGACCTCTCTATACAAGATAACCAGAGGCATCAAACTGAAATCCTCCTGTGGAAAATAAGCTAGTACTTCTGGGCCTGATGGTGTAGTGAAAACCTGTGCTTGAGGATACATTACAGTGAAAGAGCAAAGTGAATAGTAAGTAGCTATTACTTACCTCCTTAGGGAGGTGTGTTGTTTGTCTGTACATCCCCCACAGCACCTAGCACAGTACCTTGCATCTCACCTGCCACTCACTAAAAAGTCTATCAAGTTAGTTAATTATCGAGACAACGCCCTCAGAAATGAGAGAACAGTACCCTCTTATCCTTGCTGCACTTTCCAGCACTGATACGCTGCCTAAAAGAGGACTAGGGCACAGGTTTGAATTAATGTCACAAAACTGGATGGGCAAGTTACAACGGTGTTGATTAAGGAAACAGAACTCATGGTGCACCGGATATCTCCATCCTGATGAACCCTTGGAAAAATGCCAAAGATGCATATCCCCAGGCAAATGCCTGATTAGTCTGGGATTGATAGATTGGTCTAGGATTCAGCCCTACTGGGAAGATGTCTAAATTATAATCAGTGTAGAAAGCGAAGTTCTCCTAGAAGAAGAGGCAAAGGTTAAAAAGAAGAAAAGAAAAGAAAGTGAAGTCCTTTCTCCCCCAAAACCTCTCATCAATCAATCAGGGTAACAAACAGAACACTAGGGCTCTGTCTGTGGACCAAACCCAAAAGCCCTGCGGTCAGGGCCAGGAGGGTAGATCATGTGTTTGTGGCAACTTCCTCTGTGGGCTTTTGCCCAGGTCTGTCCCCAAGCATACGATGGCCAAAACTTCTGCACCAGAGCAGCATCCTGTGTAACACAGTCAGGTCCAGCAGTTAGGGAAAACTGCCCACTCAGAGTAGATAATATCTGGAAGGAATGACTGTTTGGGAAAAGTTCCAATGCTAGTTCAGTGCCAACCCTTCCCCACCTTCTCCAGCTCTCTCCCACTGGTTCCTCCCCTCTCAACTGCTCTGGTTCTTATAAAAACCTCACAGCCTTCCACTAACATCCCGTAGGAGCCTCTCTCCCTACTGCTGCTACACAAGACCCTGAGACTGACCTGCAGGACGAAACCATGAAGAGCCTGATCCTTCTTGCCATCCTGGCCGCCTTAAGCTT |
| POSTN-WT | GGTACCGAATGATTCCATTCAGAGAAATGTAAATGATAGACTAAACTCTTCCTTCCACAAAGGACCAGGGTCAGAATATAATTTAACTTTTATGCTATCTCCTCTCCAGCTCTTCTTTTGAATTGCCCCTTCATTTTCTATAACTTAATCCCAAGATTTTCATATTGAAATGGCCTCTCGATCATATTTCAGCATTTGTGGTTTCACTGTGTGCTTTCTTAATAAACTAAGCAGCAAGTTGTAGAATATTTTAATATCCCATAGTGAGTTTAAGCATAACTCAGGATACTCAGAATCCTCTCTAGTTCAGTCCAGTTCCCCTAAAGCCTTCCCAATAGAACAACAAGGAGAATTCTGACAAAATCTCTCTACCTCACCAGTGGGAAAAGAACATGGATATGACATTACAGAGGGAGCATCTTGTAGCAAAATGGATTGGAATTACACTTTAGGATGGTGTGCAGCTTGTTTATTCAAGGAAGCTGATTGTGGTTACCATTTCAGCTCATTGTTTCCCTCTGATATTCAGATTTCTTTTATCCAAAGTTTTCTGAACATTTGACATATGCATGGAGTAGTAAGAACGCACATTCCTTTGGCTAGGGATTGCATAGTGTAACATTGAAAGGCTTTAGTTATTCTTGATAAAATCAGAAGAATCATTTTGGCAATGTCCATGTTTCCTAATATGTATTTAATTGTTCTAGTTATCTCTAGATTCGTAACACTTGCTTCCAATATTGGCTGCTTTTCACCAGTGGTTATCAGTAGATGTGCTGCATAGACATGTTCAACATCACATAAAGTGTGTCATGAAAAACAATAAAGCACAAATGTTTTTCCTTTCTGCTTCATTTCAAACCTTAGAGAAATTTGTCAGAGTAGATTTATGTTAACTCTATATGTTCCTTGATATTTGAAAAAGAAAGGAAATGAGAGGTAGCTCTCCTTTTTGTACGATAGCAAGGAAAATATGGACTCTCCTACATGGTTCTTCAGCAAGTTAGAATTCTTATTTCATATCTTACACAAGTTTTAAATCTATCCAGAGTTTGTTTTTAATCAACAGCCTTTACCCCCTTGTGATTGTCAGACTCGCATCTACCTTTGTTTTCTGGTAAAAATAATAATAATAATAATCTTTCAGTTCTGATGTGAACTGCAATAACACCTAACAATAATCTTGAGCACACAGACATTATACATTCTACTCTGGAAAGGATTGCAGAATATCTCTTAAAACTCAACAAAAGAATTTTTCTTAAAAACCCTCTAAGATACAAAGGAATAAAACTGAGACTTAAACATGCAGTGAGTCAATTGTTCATATGATTAAAAATAAGTACCTTCTTTATAATGAAAAGGAAAAGTAGCTCAATGTGTTCCTTAAATATAACTAACCAAAACAAATCTTAGCTGGCAATTTGAAGTTGCCGATGCTTCCTGGAAAGAGTTCAAGCTT |
| POSTN-MU | GGTACCGAATGATTCCATTCAGAGAAATGTAAATGATAGACTAAACTCTTCCTTCCACAAAGGACCAGGGTCAGAATATAATTTAACTTTTATGCTATCTCCTCTCCAGCTCTTCTTTTGAATTGCCCCTTCATTTTCTATAACTTAATCCCAAGATTTTCATATTGAAATGGCCTCTCGATCATATTTCAGCGCCCAGTTCCCCACTGTGTGCTTTCTTAATAAACTAAGCAGCAAGTTGTAGAATATTTTAATATCCCATAGTGAGTTTAAGCATAACTCAGGATACTCAGAATCCTCTCTAGTTCAGTCCAGTTCCCCTAAAGCCTTCCCAATAGAACAACAAGGAGAATTCTGACAAAATCTCTCTACCTCACCAGTGGGAAAAGAACATGGATATGACATTACAGAGGGAGCATCTTGTAGCAAAATGGATTGGAATTACACTTTAGGATGGTGTGCAGCTTGTTTATTCAAGGAAGCTGATTGTGGTTACCATTTCAGCTCATTGTTTCCCTCTGATATTCAGATTTCTTTTATCCAAAGTTTTCTGAACATTTGACATATGCATGGAGTAGTAAGAACGCACATTCCTTTGGCTAGGGATTGCATAGTGTAACATTGAAAGGCTTTAGTTATTCTTGATAAAATCAGAAGAATCATTTTGGCAATGTCCATGTTTCCTAATATGTATTTAATTGTTCTAGTTATCTCTAGATTCGTAACACTTGCTTCCAATATTGGCTGCTTTTCACCAGTGGTTATCAGTAGATGTGCTGCATAGACATGTTCAACATCACATAAAGTGTGTCATGAAAAACAATAAAGCACAAATGTTTTTCCTTTCTGCTTCATTTCAAACCTTAGAGAAATTTGTCAGAGTAGATTTATGTTAACTCTATATGTTCCTTGATATTTGAAAAAGAAAGGAAATGAGAGGTAGCTCTCCTTTTTGTACGATAGCAAGGAAAATATGGACTCTCCTACATGGTTCTTCAGCAAGTTAGAATTCTTATTTCATATCTTACACAAGTTTTAAATCTATCCAGAGTTTGTTTTTAATCAACAGCCTTTACCCCCTTGTGATTGTCAGACTCGCATCTACCTTTGTTTTCTGGTAAAAATAATAATAATAATAATCTTTCAGTTCTGATGTGAACTGCAATAACACCTAACAATAATCTTGAGCACACAGACATTATACATTCTACTCTGGAAAGGATTGCAGAATATCTCTTAAAACTCAACAAAAGAATTTTTCTTAAAAACCCTCTAAGATACAAAGGAATAAAACTGAGACTTAAACATGCAGTGAGTCAATTGTTCATATGATTAAAAATAAGTACCTTCTTTATAATGAAAAGGAAAAGTAGCTCAATGTGTTCCTTAAATATAACTAACCAAAACAAATCTTAGCTGGCAATTTGAAGTTGCCGATGCTTCCTGGAAAGAGTTCAAGCTT |
