# Supplementary material for: RUNX1 contributes to the mesenchymal subtype of glioblastoma in a TGFβ pathway-dependent manner
Source: Cell Death Dis. 2019 Nov 21;10(12):877. doi: 10.1038/s41419-019-2108-x (PMC6872557; doi:10.1038/s41419-019-2108-x)
Supplement: Supplementary file 23 — table s4 [file 41419_2019_2108_MOESM23_ESM.docx]

Table S4. Association of RUNX1 expression with various clinical parameters in CGGA GBM samples.

| CGGA | n | Low RUNX1 expression | High RUNX1  expression | P Value |
| --- | --- | --- | --- | --- |
| Age |  |  |  |  |
| ≤40 | 39 | 24 | 15 | 0.12 |
| >40 | 89 | 40 | 49 |  |
| Gender |  |  |  |  |
| female | 48 | 24 | 24 | 1 |
| male | 80 | 40 | 40 |  |
| GBM |  |  |  |  |
| primary | 109 | 58 | 51 | 0.13 |
| secondary | 19 | 6 | 13 |  |
| Total | 128 | 64 | 64 |  |
