# Supplementary material for: RUNX1 contributes to the mesenchymal subtype of glioblastoma in a TGFβ pathway-dependent manner
Source: Cell Death Dis. 2019 Nov 21;10(12):877. doi: 10.1038/s41419-019-2108-x (PMC6872557; doi:10.1038/s41419-019-2108-x)
Supplement: Supplementary file 24 — table s5 [file 41419_2019_2108_MOESM24_ESM.docx]

Table S5. Characteristics of patients

| Patient | TBD0207 | TBD0220 |
| --- | --- | --- |
| Gender | Male | Male |
| Age | 52 | 46 |
| Tumor location | Left Parietal lobe | Left Temporal lobe |
| Tumor Grade | GBM | GBM |
| Survival(in months) | alive | 11 |
| Ki-67(%) | 30 | 50 |
