# Supplementary material for: RUNX1 contributes to the mesenchymal subtype of glioblastoma in a TGFβ pathway-dependent manner
Source: Cell Death Dis. 2019 Nov 21;10(12):877. doi: 10.1038/s41419-019-2108-x (PMC6872557; doi:10.1038/s41419-019-2108-x)
Supplement: Supplementary file 25 — table s6 [file 41419_2019_2108_MOESM25_ESM.docx]

Table S6.

Sequences of siRNA and shRNA

|  | Binding Sequence 5’---3’ |
| --- | --- |
| ShRUNX1-1 | GACAGCATATTTGAGTCATTT |
| ShRUNX1-2 | CTTGGAATGAATCCTTCTAGA |
| ShRUNX1-3 | CCAGGTTGCAAGATTTAATGA |
| ShRUNX1-4 | GACATCGGCAGAAACTAGATG |
|  | Sequence |
| siSUV39H1 | GCCUGAGAAAUGACAGACUTT |
| siSMAD3 | CAGAAGAATGGTACAAATCCAAG |
